# Mechanisms of ligand binding

**DOI:** 10.1063/5.0020997

**Published:** 2020-11-16

**Authors:** Enrico Di Cera

**Affiliations:** Edward A. Doisy Department of Biochemistry and Molecular Biology, Saint Louis University School of Medicine, St. Louis, Missouri 63104, USA

## Abstract

Many processes in chemistry and biology involve interactions of a ligand with its molecular target. Interest in the mechanism governing such interactions has dominated theoretical and experimental analysis for over a century. The interpretation of molecular recognition has evolved from a simple rigid body association of the ligand with its target to appreciation of the key role played by conformational transitions. Two conceptually distinct descriptions have had a profound impact on our understanding of mechanisms of ligand binding. The first description, referred to as induced fit, assumes that conformational changes follow the initial binding step to optimize the complex between the ligand and its target. The second description, referred to as conformational selection, assumes that the free target exists in multiple conformations in equilibrium and that the ligand selects the optimal one for binding. Both descriptions can be merged into more complex reaction schemes that better describe the functional repertoire of macromolecular systems. This review deals with basic mechanisms of ligand binding, with special emphasis on induced fit, conformational selection, and their mathematical foundations to provide rigorous context for the analysis and interpretation of experimental data. We show that conformational selection is a surprisingly versatile mechanism that includes induced fit as a mathematical special case and even captures kinetic properties of more complex reaction schemes. These features make conformational selection a dominant mechanism of molecular recognition in biology, consistent with the rich conformational landscape accessible to biological macromolecules being unraveled by structural biology.

## I. INTRODUCTION

The binding of a ligand to its target is central to many processes in chemistry and biology. For this reason, theoretical treatments of the equilibrium and kinetic underpinnings of ligand binding have occupied researchers for well over a century. The first attempt to rationalize the reversible encounter of a ligand with a biological macromolecule to produce a complex was proposed by Fischer in 1894^1^ as a lock-and-key mechanism of recognition, envisioning a nearly perfect shape complementarity between the ligand and its binding site. Structural rigidity of the ligand and its target dictate the rules for specificity in this mechanism and explain changes in affinity among different ligands binding to the same target or of the same ligand binding to different targets. Fischer's original idea remains relevant to this day in drug design, where pharmacological leads are progressively rigidified to promote shape complementarity with their biological target.^2^ It took several decades for the scientific community to realize that biological macromolecules are intrinsically flexible and may affect the energetics of ligand recognition by changing the shape of the binding site. In 1951, Wyman and Allen proposed a radical new explanation for hemoglobin's Bohr effect, i.e., the linkage between pH and oxygen binding, based on alternative conformations accessible to the protein.^3^ Later on, Eigen envisioned multiple preexisting states for a host controlling ionic interactions in solution^4^ and Koshland extended the lock-and-key mechanism to account for a conformational rearrangement following the initial encounter to produce a more stable complex through a mechanism that he called “induced fit.”^5^ Development of the allosteric concept^6,7^ soon led to the celebrated Monod-Wyman-Changeux model of protein allostery based on a preexisting equilibrium between alternative conformations with different ligand binding affinity.^8^ The elegance of this model stems from the complexity of functional behaviors and cooperativity made possible by a simple linkage established between ligand binding and conformational transitions. An equally elegant model developed by Koshland, Nemethy, and Filmer envisioned linkages with conformational transitions taking place at each step of ligation and through nearest-neighbor interactions for multimeric proteins.^9^ The advent of X-ray structural biology and nuclear magnetic resonance (NMR) has firmly established the conformational plasticity of biological macromolecules^10,11^ and its relevance to drug design.^12^ Valuable extensions of the original allosteric concept have been presented^13–17^ and now include a dynamic view of protein conformations generated by folding and binding along an ensemble of accessible states.^11,18–21^ The current challenge for the experimentalist is to decipher the signatures of such emerging complexity from analysis of experimental data. The goal is to produce a coherent description of ligand binding that is consistent with both structural and functional data.

This review focuses on the analysis and interpretation of the kinetics of ligand binding mechanisms that are key to establishing a correlation with structure and any linked conformational transition. Our treatment deals with the most common situation encountered in practice, i.e., the reaction of a ligand with a single site on its target. Emphasis is given to the mathematical underpinnings of this interaction when studied through its kinetic components. We show that even this simple interaction may give rise to substantial functional complexity through the interplay of ligand binding and conformational transitions. A rigorous understanding of this complexity is critical for the correct interpretation of experimental data, the validation of structural information, and to advance basic knowledge and translational applications. Our discussion complements and updates excellent contributions available through monographs^22–25^ and reviews^26–29^ that should be consulted to expand on the topics presented here.

## II. ONE STEP REACTION MECHANISM

The phenomenological approach to ligand binding to a biological macromolecule at equilibrium, in a closed system under conditions of constant temperature and pressure, is based on the formulation of a partition function as a polynomial expansion in the ligand activity x of degree N, equal to the number of binding sites.^25,30^ All relevant thermodynamic quantities are related to this “binding polynomial”^31,32^ by simple transformations and offer a rigorous interpretation of the underlying energetics, yet provide little insight into the molecular mechanism of recognition. This is because the polynomial takes the same general form once the value of N is defined, regardless of the number of conformations involved. The kinetic treatment of ligand binding to a biological macromolecule depends not only on the number of distinct ligated species in the system but also on their conformations. Both ligation and conformational states define the dimensionality of the system and contribute to the possible reaction trajectories.^24^ Unlike equilibrium, kinetics may find differences among various mechanisms even for systems that share the same value of ligand binding sites, N. Hence, the mathematical treatment of the kinetic properties of a system can be quite complex, even for the simplest case of ligand binding to a biological macromolecule containing a single site ( N = 1). From this complexity comes the ability to infer precious information on the mechanism of ligand binding that is not possible to extract from the equilibrium treatment.

We start our discussion with a reaction scheme describing the transition of a system between two states, i.e.,

$$E_{1}\begin{array}{c} k_{12}\\ ⇄\\ k_{21} \end{array}E_{2}.$$ (1)

For the sake of clarity, we shall focus on the case where the “system” represents a biological macromolecule and the “states” refer to distinct conformations, e.g., folded and unfolded, or open and closed. The reaction in Eq. (1) is completely defined by the first-order rate constants $k_{12}$ and $k_{21}$, measured in s^−1^, reflecting the forward and reverse transitions between $E_{1}$ and $E_{2}$. Under the assumptions that the system is closed and the macromolecule does not change its aggregation state, the time evolution of the two species in Eq. (1) is given by the differential equations

$$\left(\begin{array}{c} {dE}_{1}/dt\\ {dE}_{2}/dt \end{array}\right)=\left(\begin{array}{cc} {-k}_{12} & k_{21}\\ k_{12} & -k_{21} \end{array}\right)\left(\begin{array}{c} E_{1}\\ E_{2} \end{array}\right).$$ (2)

Equilibrium is reached in the limit t→∞ where the ratio

$$K_{12}=\frac{k_{12}}{k_{21}}=\frac{E_{2}\left(\infty \right)}{E_{1}\left(\infty \right)}$$ (3)

defines the dimensionless equilibrium constant, $K_{12}$, as the ratio of the populations of $E_{2}$ and $E_{1}$ at t=∞. The value of $K_{12}$ can be the same for different combinations of $k_{12}$ and $k_{21}$, so long as their ratio remains unchanged. Hence, information on the equilibrium properties of the system depends on a single independent constant, $K_{12}$, from which the two independent rate constants, $k_{12}$ and $k_{21}$, that define how the macromolecule transitions between $E_{1}$ and $E_{2}$ cannot be resolved. Once the value of $K_{12}$ is known, the fraction of macromolecules in-state, $E_{1}$ and $E_{2}$, can be calculated as

$$f_{1}=\frac{k_{21}}{k_{12}+k_{21}}=\frac{1}{{1+K}_{12}}$$ (4a)

$$f_{2}=\frac{k_{12}}{k_{12}+k_{21}}=\frac{K_{12}}{{1+K}_{12}}.$$ (4b)

The information conveyed by $f_{1}$ and $f_{2}$ is purely phenomenological and states which of the two conformations, $E_{1}$ or $E_{2}$, is more populated at equilibrium as a result of $k_{12}$ being faster than $k_{21}$, or vice versa. The exact values of $k_{12}$ and $k_{21}$ remain undetermined and so is the timescale of the transitions $E_{1}\to E_{2}$ and $E_{1}\leftarrow E_{2}$ defined as the inverse value of $k_{12}$ and $k_{21}$.^22,28^ An additional relationship between $k_{12}$ and $k_{21}$ is derived from measurements of the time-dependent evolution of the system, i.e.,

$$E_{1}\left(t\right)=E_{1}\left(\infty \right)+\left[E_{1}\left(0\right)-E_{1}\left(\infty \right)\right]e^{-\alpha t}$$ (5a)

$$E_{2}\left(t\right)=E_{2}\left(\infty \right)+\left[E_{2}\left(0\right)-E_{2}\left(\infty \right)\right]e^{-\alpha t},$$ (5b)

where $E_{i}\left(\infty \right)$ and $E_{i}\left(0\right)$ are the values of $E_{i}\left(t\right)$ at t=∞ and t=0, respectively ( i=1, 2). The quantity

$$\alpha =k_{12}+k_{21}$$ (6)

defines the rate of relaxation to equilibrium and is equal to the non-zero eigenvalue, with reversed sign, of the matrix of rate constants in Eq. (2). The symmetry of Eq. (6) is noteworthy. Regardless of whether $k_{12}$ is faster or slower than $k_{21}$, equilibrium is always attained with the same rate, or within the same timescale defined as the inverse value of α. Furthermore, the rate of approach to equilibrium, α, is always faster than the fastest individual transitions $k_{12}$ and $k_{21}$,^22–24^ i.e., the rate at which $E_{1}$ and $E_{2}$ reach equilibrium is always faster than the individual forward and reverse transitions $E_{1}\to E_{2}$ and $E_{1}\leftarrow E_{2}$. However, as seen in the case of the equilibrium constant $K_{12}$, the value of α is a single number from which the individual rate constants $k_{12}$ and $k_{21}$ cannot be resolved unambiguously. Knowledge of both $K_{12}$ and α is necessary to resolve the rates for the individual transitions $E_{1}\to E_{2}$ and $E_{1}\leftarrow E_{2}$ and confirm the validity of the mechanism in Eq. (1) through consistency of Eqs. (3) and (6).

The foregoing example details basic differences between equilibrium and kinetics treatments for the scheme in Eq. (1), but shows that both approaches are necessary to resolve the underlying rate constants. Consider next the case where the two-state reaction scheme in Eq. (1) is perturbed by a dimensionless factor δ that changes the rate of the $E_{1}\to E_{2}$ transition. Analogous perturbation of the reverse $E_{1}\leftarrow E_{2}$ transition obeys the same mathematical treatment because of the intrinsic symmetry of Eq. (1). The relevant reaction scheme becomes

$$E_{1}\begin{array}{c} k_{12}\delta \\ ⇄\\ k_{21} \end{array}E_{2},$$ (7)

and the rate of relaxation to equilibrium is

$$\alpha (\delta )=k_{12}\delta +k_{21}.$$ (8)

**FIG. 1.**
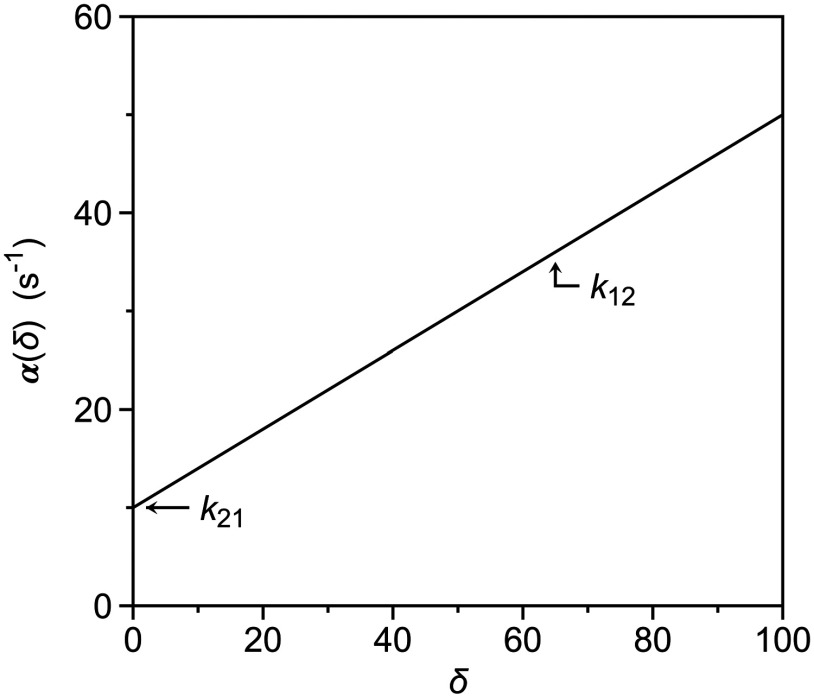
Dependence of the rate of relaxation to equilibrium, $\alpha \left(\delta \right)$, as a function of the perturbation factor δ. The plot is a straight line from which the individual rate constants of the mechanism in Eq. (7) can be resolved as the slope ( $k_{12}$) and intercept ( $k_{21}$).

Introduction of the perturbation generates a strategy to resolve the individual rate constants $k_{12}$ and $k_{21}$ from measurements of α(δ) as a function of δ (Fig. 1). The resulting plot is a straight line, with slope $k_{12}$ and intercept $k_{21}$. Knowledge of these independent rate constants also defines the value of the equilibrium constant $K_{12}$ through Eq. (3) and makes independent measurement of this parameter only needed to confirm validity of the kinetic experiment. In principle, a method that measures the value of α under the effect of a perturbation δ will be able to resolve the independent rate constants defining the mechanism in Eq. (1).

Consider the case where the perturbation factor δ in Eq. (7) measures the activity or concentration x of a ligand X binding to a biological macromolecule E to generate the complex EX. For the sake of simplicity, we assume that the ligand is in large excess over the macromolecule and that its concentration does not change significantly as a result of the binding interaction. This is the so-called pseudo-first-order approximation and describes the conditions most commonly encountered in practice.^22,23^ We also assume that the ligand binds to a single site and that binding does not change the aggregation state of the macromolecule. Under these assumptions, Eq. (7) describes binding of a ligand to a biological macromolecule according to the scheme

$$E_{\;}\begin{array}{c} k_{on}x\\ ⇄\\ k_{\mathrm{off}} \end{array}EX.$$ (9)

The rate constant $k_{on}$ has dimensions of M^−1^s^−1^ and is no longer first-order as in Eq. (1) or (7) but second-order because the perturbation factor depends on the concentration of the ligand. The rate constant $k_{\mathrm{off}}$ retains its first-order dimensionality in s^−1^ and measures the rate of dissociation of the complex EX into the parent species E and X. Unlike $k_{12}$ and $k_{21}$ in Eq. (1) or (7), the values of $k_{on}$ and $k_{\mathrm{off}}$ have different dimensionality and cannot be compared directly. Additional distinctions must be considered. Processes that are first-order, as those described in Eq. (1), are minimally affected by translational and rotational diffusion that is extremely important in the second-order rate constant of association for binding interactions.^27^ This is a reason why biological interactions should be studied in solution with techniques like stopped flow,^33^ temperature jump,^4^ or NMR,^33^ rather than surface plasmon resonance where the macromolecule or the ligand are immobilized to a matrix.^34^ Elimination of translational and rotational degrees of freedom may bias the measured rate constant for ligand association and may return incorrect values for the equilibrium binding constant. Additional differences between first-order and second-order rate constants is that the latter tend to be extremely sensitive to electrostatics and solution conditions, especially salt concentration.^26^ Information on the values of $k_{on}$ and $k_{\mathrm{off}}$ is critical to arrive at the equilibrium dissociation constant $K_{d}=\frac{k_{\mathrm{off}}}{k_{on}}$.^30^ The value of $K_{d}$ defines the strength of interaction and establishes the relative affinity of different ligands binding to the same target or of the same ligand binding to different targets. Changes in $K_{d}$ also give information on how the interaction is affected by changes in solution conditions, temperature, pressure, or mutations introduced in the system.^30^ Although this is relevant information about the properties of the system, the value of $K_{d}$ provides a rather “static” interpretation of the interaction. Information of major biological and biophysical interest is contained in the individual rate constants $k_{on}$ and $k_{\mathrm{off}}$. Importantly, the value of $k_{on}$ is limited by diffusion to about 6.5 × 10^8^ M^−1^s^−1^ under physiological conditions,^27,35^ and each system has optimized this value based on evolutionary needs of increased affinity and speed. Relevant examples are values of $k_{on}$ in the 10^6^–10^8^ M^−1^s^−1^ range observed in toxins that neutralize the function of ion channels^36^ or neural transmission^37^ to cause paralysis in the prey and inhibitors of coagulation factors that cause the prey to bleed to death^38^ or rapidly neutralize the action of nucleases in degrading RNA.^27,39^ Binding affinity can also be optimized by reducing the value of $k_{\mathrm{off}}$, especially when the value of $k_{on}$ has maxed out. Although this may not be convenient in signaling pathways that require fast rates of association and rapid switch of on and off signals,^40^ it often becomes desirable in drug design to increase the so-called “residence time” of a therapeutic molecule on its target.^28^ Indeed, successful drug design is a balancing act between optimizing $k_{on}$ for rapid association and limiting $k_{\mathrm{off}}$ to increase stability of the complex with the biological target. In general, when molecules compete for the same target, binding at the diffusion controlled limit provides a source of selection, as shown convincingly by the binding of various psychoactive substances to the membrane transporters for the monoamines serotonin and dopamine,^41^ but so does modulation of $k_{\mathrm{off}}$ among competing ligands that have optimized their $k_{on}$.^42^

**FIG. 2.**
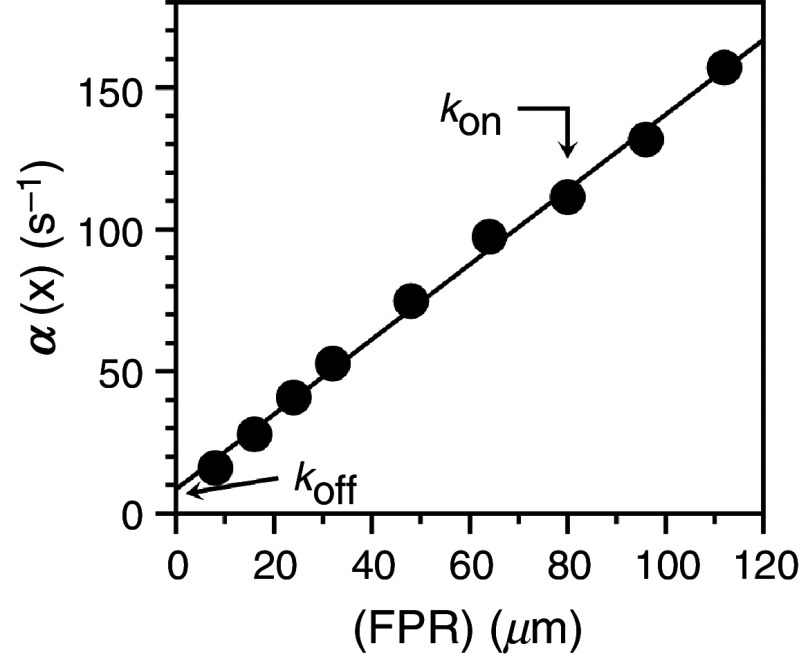
Rapid kinetics of FPR binding to the thrombin mutant D194A.^50^ The straight line was drawn according to Eq. (10) with best-fit parameter values $k_{on}$ = 1.3 ± 0.1 *μ*M^−1^s^−1^ and $k_{\mathrm{off}}$ = 8.5 ± 0.1 s^−1^. The plot is consistent with a lock-and-key mechanism of interaction [Eq. (9)] between the tripeptide FPR and the thrombin mutant. Experimental conditions are: 400 mM ChCl, 50 mM Tris, 0.1% PEG8000, pH 8.0 at 15 °C.

The reaction scheme in Eq. (9) offers the simplest possible interpretation of a binding interaction in terms of the so-called lock-and-key mechanism as envisioned by Fischer back in 1894^1^ at a time when biological macromolecules were thought of as rigid bodies, in contrast to the current view that they are inherently dynamic.^10,43–48^ In this mechanism, ligand and its biological target are preconfigured for optimal binding through a rigid body association. When the properties of the system are studied in the approach to equilibrium using an apparatus for rapid kinetics, the relaxation obeys a straight line, as a function of the ligand concentration x and the values of $k_{on}$ and $k_{\mathrm{off}}$ are the slopes and intercept of the plot, i.e.,

$$\alpha (x)=k_{on}x+k_{\mathrm{off}},$$ (10)

which is analogous to Eq. (8). We stress that the range of processes that can be studied experimentally through relaxation kinetics is limited by the time resolution of the system. Another important limiting factor is that the processes accessible to experimental measurements are always the slowest ones in the reaction, relative to the resolution of the instrument. Any conformational transition [Eq. (1)] or binding interaction [Eq. (9)] that takes place on a timescale faster than the range of detection of the instrument will be undetected. A stopped flow is restricted by the dead time of the instrument to values < 500 s^−1^ but a continuous flow apparatus can detect much faster rates, in the 2000–20 000 s^−1^ range.^49^ Even faster ranges in the 10^5^–10^6^ s^−1^ range can be detected by temperature jump if the reaction is linked to sufficient perturbation of affinity linked to temperature changes.^4,15^ An example of the plot in Eq. (10) is given in Fig. 2 for the case of the tripeptide H-D-Phe-Pro-Arg-p-nitroanilide (FPR) binding to the active site of the mutant D194A of the clotting protease thrombin.^50^ The binding mechanism is consistent with a lock-and-key, rigid body type of association with values of $k_{on}$ = 1.3 ± 0.1 × 10^6^ M^−1^s^−1^ in the diffusion-limited rate range and a relatively fast $k_{\mathrm{off}}$= 8.5 ± 0.5 s^−1^, corresponding to a value of the equilibrium constant $K_{d}$ = 6.5 ± 0.6 *μ*M. There are several interactions in biology that obey Eq. (9) and give a linear plot of the relaxation to equilibrium as shown in Fig. 2. They are particularly relevant to drug design where binding is optimized by restricting the conformational landscape of the ligand.^2,28,51^ Conformational changes may pose challenges in the design of optimal inhibitors of biological targets.^12^ However, the widely accepted importance of protein flexibility in biomolecular recognition suggests increasing target flexibility in the bound state by ligand design as a new strategy for drug discovery.^52^

## III. TWO STEP REACTION MECHANISM: THE RAPID EQUILIBRIUM APPROXIMATION

The lock-and-key mechanism assumes that the ligand and its target interact without any linked conformational transitions and offers a valuable reference point for the interpretation of more complex binding mechanisms. Whenever binding is involved, there will be a relaxation that increases linearly with the ligand concentration as predicted by Eq. (10). This conclusion holds true regardless of the complexity of the reaction mechanism. As early as 1951, Wyman and Allen drew attention to conformational changes as possible driving forces for binding and linkage effects in hemoglobin,^3^ and set in motion a conceptual revolution that would culminate with formulation of the celebrated allosteric concept a few years later.^6–8^ Conformational changes linked to binding are also relevant to enzyme kinetics when investigating the microscopic pathway for ligand binding in the kinetic mechanism and the overall rate to form the active Michaelis complex. When the binding step is rate-limiting for catalysis, it will depend on the viscosity of the solution. The lack of such dependence suggests the presence of a conformational change and raises the question as to whether it follows or precedes the binding step. The two possible cases linking the basic lock-and-key mechanism describing binding [Eq. (9)] with an elementary conformational transition [Eq (1)] are discussed below. In the first case, the conformational change follows the binding step and defines the so-called induced fit (IF) mechanism first proposed by Koshland.^5^ In the second case, the conformational change precedes the binding step and defines the so-called pre-equilibrium mechanism first proposed by Eigen^4^ and later on cast in a more structural context^44^ as conformational selection (CS). Under the assumptions used for the lock-and-key mechanism in Eq. (9), the relevant reaction scheme for IF is

$$E\begin{array}{c} k_{on}x\\ ⇄\\ k_{\mathrm{off}} \end{array}EX\begin{array}{c} k_{23}\\ ⇄\\ k_{32} \end{array}E\prime X.$$ (11)

The first step in the reaction scheme is identical to the lock-and-key mechanism [Eq. (9)]. The second step describes a conformational rearrangement of the complex into a new final state and is analogous to the two-state reaction scheme in Eq. (1). The relevant reaction scheme for CS is

$${E^{*}}_{\;}\begin{array}{c} k_{12}\\ ⇄\\ k_{21} \end{array}E_{\;}\begin{array}{c} k_{on}x\\ ⇄\\ k_{\mathrm{off}} \end{array}EX.$$ (12)

The first step describes a conformational rearrangement analogous to Eq. (1) that preexists in the biological macromolecule before the binding event, and the second step is identical to the lock-and-key mechanism [Eq (9)]. Both IF and CS merge a basic one-step mechanism involving binding [Eq. (9)] and conformational transitions [Eq. (1)] but in reverse order. A challenge for the experimentalist is to establish which mechanism, IF or CS, is at play and how the rates describing the conformational changes can be measured from analysis of experimental data.

The two schemes in Eqs. (11) and (12) feature a similar topology where two one-step reactions are arranged in reversed order. This presages very different kinetics for IF and CS and suggests that the two mechanisms are intrinsically distinct and mathematically irreducible. A number of considerations support such a conclusion. When the ligand concentration decreases ( x→0), IF collapses toward a single conformation E, whereas CS defines the $E^{*}⇄\;E$ preexisting equilibrium between the two possible conformations of the macromolecule. The opposite happens when the ligand concentration increases ( x→∞): IF defines the $EX\;⇄\;E^{\prime }X$ equilibrium between the two possible conformations of the complex whereas CS collapses toward the single conformation EX. The number of conformations linked to binding progressively increases for IF but decreases for CS, i.e., IF generates new conformations as a result of the binding interaction but CS reduces the number of conformations from the preexisting ones when binding occurs. There are three species that define Eqs. (11) and (12). Because the system is closed and mass is conserved, only two of these species are independent, and there are only two independent non-zero relaxations associated with each reaction scheme: one reflects binding and eventually increases linearly with x as seen for Eq. (10), the other reflects conformational transitions and features a distinct behavior for IF and CS. In the former case, the rate associated with the conformational transition increases with x as the macromolecule transitions from a single free conformation E to an equilibrium between two bound conformations $EX\;⇄\;\;E^{\prime }X$. In the latter case, the opposite is observed, and the rate associated with the conformational transition decreases with x as the macromolecule goes from an equilibrium between two free conformations $E^{*}⇄\;\;E$ to a single bound conformation EX. These are intuitive expectations about the kinetics of IF and CS based on simple inspection of the topology of the two reaction schemes in Eqs. (11) and (12). They imply that IF and CS are mutually exclusive mechanisms of recognition, offering distinct and irreducible interpretations of the linkage between binding and conformational transitions.^28,53^

Support for the foregoing conclusion comes from analysis of the functional behavior of the two mechanisms when binding takes place on a significantly faster timescale than conformational transitions, i.e., under the so-called “rapid equilibrium approximation.”^22,23^ The condition can be understood by comparing the rates of relaxation to equilibrium for a conformational transition [Eq. (6)] and binding [Eq. (10)]. The rate is constant for the former process but increases linearly with the ligand concentration for the latter. At high enough values of x, binding will always be faster than any linked conformational change. When the rate of ligand dissociation $k_{\mathrm{off}}$ is faster than the rates associated with conformational transitions, binding will reach equilibrium faster than the associated conformational change, and the two relaxations associated with IF and CS become separated by widely different time scales. Binding, if not too fast to detect by a stopped flow apparatus, will follow a straight line, according to Eq. (10). The conformational change will unfold over a slow timescale, typically within the range of detection of the stopped flow apparatus. Under these conditions, the two schemes in Eqs. (11) and (12) contract into

$$\left\langle \langle E|EX\rangle \right\rangle \begin{array}{c} k_{23}\\ ⇄\\ k_{32} \end{array}E\prime X$$ (13a)

$${E^{*}}_{\;}\begin{array}{c} k_{12}\\ ⇄\\ k_{21} \end{array}{\left\langle \langle E|EX\rangle \right\rangle }_{.}$$ (13b)

The term $\left\langle \langle E|EX\rangle \right\rangle$ denotes an equilibrium distribution of free and bound species. The “contracted” reaction schemes in Eqs. (13a) and (13b) are equivalent to a two-state reaction [Eq. (1)] involving a conformational exchange between two bound forms for IF [Eq. (13a)] or two free forms for CS [Eq. (13b)]. Of the two species in Eqs. (13a) and (13b), only one is independent, and the kinetics of the systems is governed by a single relaxation rate

$$\alpha \left(x\right)=k_{23}\frac{x}{K_{d}+x}+k_{32}=k_{23}f+k_{32}$$ (14a)

$$\alpha \left(x\right)=k_{12}+k_{21}\frac{K_{d}}{K_{d}+x}=k_{12}+k_{21}(1-f),$$ (14b)

where f is the fractional saturation of the macromolecule within the $\left\langle \langle E|EX\rangle \right\rangle$ equilibrium [see also Eqs. (4a) and (4b)]. It is important to recognize the similarity of Eqs. (14a) and (14b) with Eq. (8). In the case of IF, the value of $k_{23}$ is perturbed by the fraction of macromolecules in the bound form within the $\left\langle \langle E|EX\rangle \right\rangle$ equilibrium. In the case of CS, the value of $k_{21}$ is perturbed by the fraction of macromolecules in the free form within the $\left\langle \langle E|EX\rangle \right\rangle$ equilibrium. The expressions in Eqs. (14a) and (14b) reduce the independent parameters in Eqs. (11) and (12) from four to three and enable resolution of all relevant constants from analysis of a single relaxation. The relaxation is saturable, and the three independent parameters are resolved as the limiting values at high and low ligand concentration, plus the mid-point of the transition between these values. Another attractive feature of the simplification generated by the rapid equilibrium approximation is that IF and CS can easily be distinguished from analysis of experimental data because they make very different predictions about the properties of the slow relaxation. The expression associated with IF [Eq. (14a)] predicts a saturable hyperbolic *increase* in α(x) with x, from a value of $k_{32}$ to a plateau of $k_{23}+k_{32}$. The expression associated with CS [Eq. (14b)] predicts a saturable hyperbolic *decrease* in α(x) with x, from a value of $k_{12}+k_{21}$ to a plateau of $k_{12}$. The kinetics of a large number of systems have been interpreted using the rapid equilibrium approximation. In turn, the prevalence of systems featuring a saturable relaxation increasing hyperbolically with x has fostered the notion that IF is the dominant mechanism of molecular recognition in biology, with CS documented only in a handful of cases.^28^

**FIG. 3.**
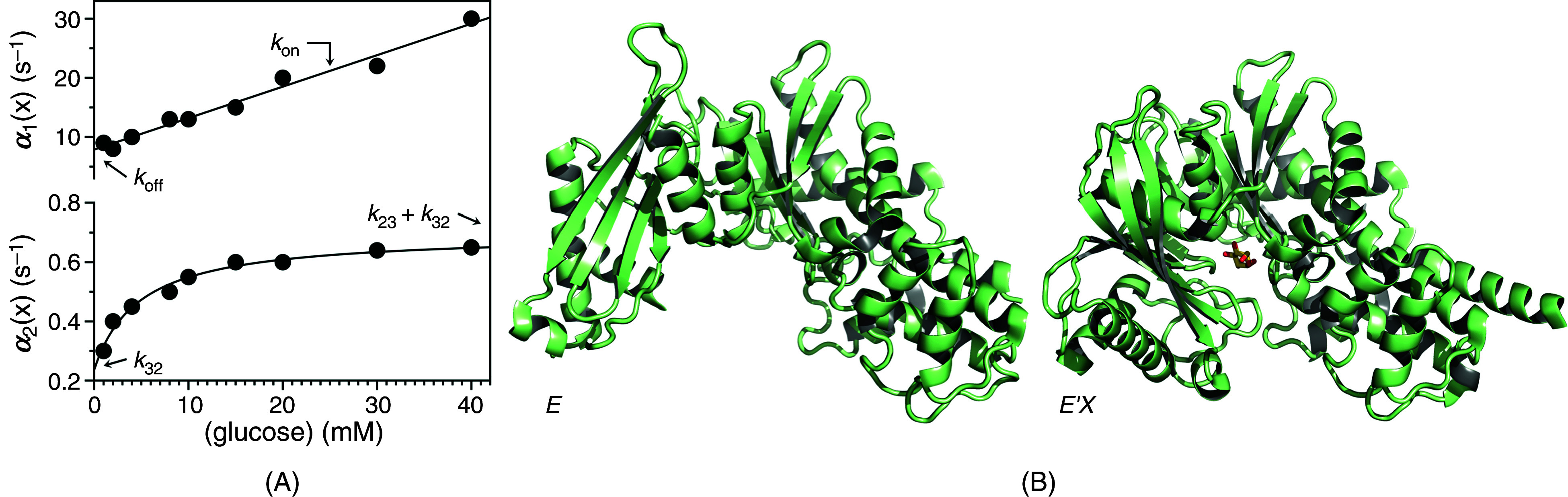
(A) Rates of relaxation for glucose binding to glucokinase.^54^ The original report used the rapid equilibrium approximation to assign the mechanism as IF [Eq. (13a)] because the two relaxations are widely separated. Interpretation of the fast relaxation according to Eq. (10) yields best-fit parameter values $k_{on}$ = 0.53 ± 0.02 mM^−1^s^−1^ and $k_{\mathrm{off}}$ = 7.9 ± 0.5 s^−1^, with a predicted $K_{d}$ = 15 ± 1 mM. Analysis of the slow relaxation according to Eq. (14a) gives best-fit parameter values $k_{23}$ = 0.24 ± 0.01 s^−1^, $k_{32}$ = 0.46 ± 0.02 s^−1^, and $K_{d}$ = 4.7 ± 0.4 mM, which is threefold different from the value predicted by the fit of the fast relaxation. The relevant parameters of IF are indicated in the plot as the intercept ( $k_{\mathrm{off}}$) and slope ( $k_{on}$) for the fast relaxation, and the lower ( $k_{32}$) or upper ( $k_{23}^{\;}+k_{32}$) limits for the slow relaxation. The mid-point of the transition in the slow relaxation gives a value of $K_{d}$ that differs from that predicted by the fast relaxation. (B) Crystal structures of glucokinase free and bound to glucose used in support of the IF mechanism [Eq. (11)]. The free form (right) assumes an open conformation^55^ and binding of glucose induces a large conformational change (left) that closes the active site region around the ligand.^56^ Panel (A) adapted with permission from Ref. 59.

As an example of a biologically relevant system obeying the rapid equilibrium approximation, we discuss the case of glucose binding to glucokinase.^54^ Rapid kinetics of glucose binding to the enzyme obey two relaxations over widely separated time scales [Fig. 3(A)]. The fast relaxation increases linearly with glucose concentration with values of $k_{on}$ = 0.53 ± 0.04 × 10^3^ M^−1^s^−1^ much smaller than the diffusion-limited rate and $k_{\mathrm{off}}$ = 7.9 ± 0.8 s^−1^, corresponding to a value of the intrinsic equilibrium dissociation constant $K_{d}$= 15 ± 1 mM. The slow relaxation increases hyperbolically with the glucose concentration from a value of $k_{32}$ = 0.24 ± 0.02 s^−1^ to the sum $k_{23}+k_{32}$= 0.46 ± 0.04 s^−1^. In this interpretation, glucose binds to glucokinase with a very low affinity and then induces a slow conformational change that optimizes the initial encounter into a final complex. IF causes a strengthening of the initial interaction with a resulting apparent equilibrium dissociation constant

$$K_{d,\;\mathrm{app}}=\frac{E\left(\infty \right)}{EX\left(\infty \right)+E\prime X\left(\infty \right)}x=\frac{{k_{\mathrm{off}}k}_{32}}{{k_{on}k}_{32}+{k_{on}k}_{23}}=K_{d}\frac{k_{32}}{k_{32}+k_{23}}$$ (15)

that is lower than the intrinsic $K_{d}$ measured as the ratio $K_{d}=\frac{k_{\mathrm{off}}}{k_{on}}$. The value of $K_{d,\mathrm{app}}$ would be measured from titration of glucokinase with glucose at equilibrium and would overestimate the affinity of the initial encounter by a factor of 2. When a binding interaction is interpreted in terms of IF, the affinity measured at equilibrium is the result of an optimized fit and always overestimates the affinity of the initial encounter. Evidence from structural biology has been used to support the IF mechanism for glucokinase [Fig. 3(B)]: the free form assumes an open conformation^55^ and binding of glucose induces a large conformational change that closes the active site region around the ligand.^56^

**FIG. 4.**
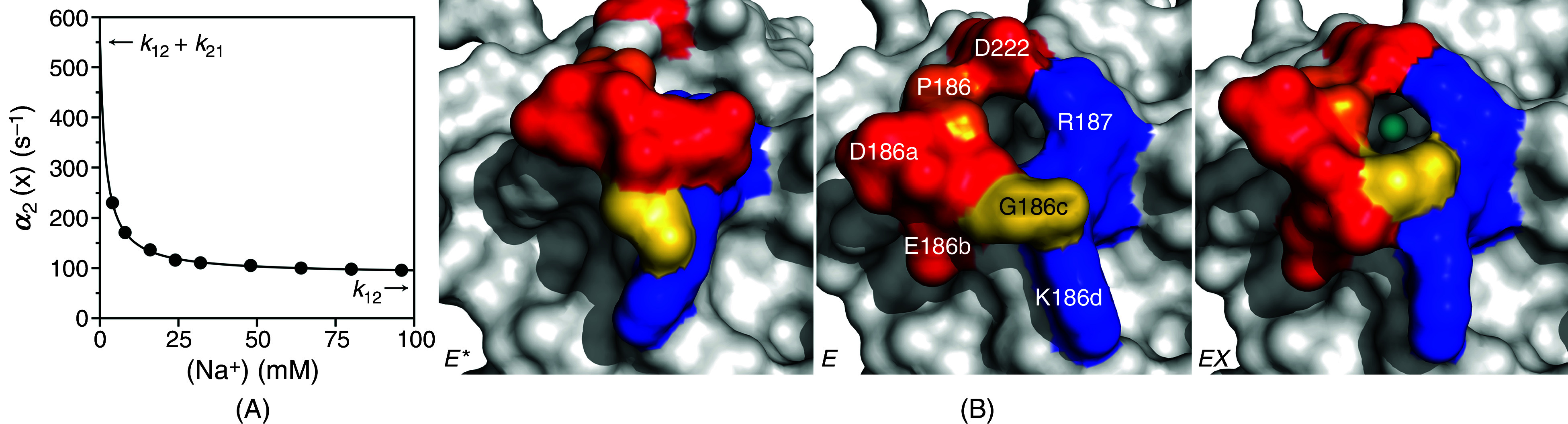
(A) Rate of relaxation for Na^+^ binding to the thrombin mutant S195A. For this interaction, only a slow relaxation decreasing with ligand concentration could be measured experimentally, providing direct and unequivocal support for the mechanism of CS [Eq. (12)]. Analysis of the slow relaxation according to Eq. (14b) gives best-fit parameter values $k_{12}$ = 88 ± 7 s^−1^, $k_{21}$ = 480 ± 30 s^−1^ and $K_{d}$ = 1.7 ± 0.1 mM. The relevant parameters of CS are indicated in the plot as the limits for high ( $k_{12}$) and low ( $k_{12}^{\;}+k_{21}$) ligand concentration. The mid-point of the transition gives the value of $K_{d}$. Experimental conditions are: 400 mM ChCl, 50 mM Tris, 0.1% PEG8000, pH 8.0, at 25 °C. (B) Crystal structures of thrombin reveal two conformations of the pore of entry of Na^+^ defined by residues in the 180 and 220 loops. The pore is rendered in surface representation with relevant residues labeled (middle) and colored according to their electrostatic properties (blue: positively charged; red: negatively charged; orange: hydrophobic; yellow: neutral). In the free form (left and middle), the pore is in equilibrium between open (middle)^57^ and closed (left)^58^ forms. Binding of Na^+^ (cyan ball) takes place in the open form of the pore (right) with minimal conformational changes.^57^

The case of Na^+^ binding to the clotting protease thrombin offers a relevant example of CS under the rapid equilibrium approximation. In this case, the fast relaxation pertaining to Na^+^ binding is too fast to measure by stopped flow and requires alternative experimental approaches.^49^ The slow relaxation can be measured by stopped flow and decreases hyperbolically with the Na^+^ concentration from the sum $k_{12}+k_{21}$ = 570 ± 40 s^−1^ to the value of $k_{12}$ = 88 ± 7 s^−1^ [Fig. 4(A)]. Binding takes place to only one of two alternative conformations of the Na^+^ site that preexists in equilibrium and with a value of the equilibrium constant $K_{d}$ =1.7 ± 0.1 mM. Structural biology supports the CS mechanism for Na^+^ binding to thrombin [Fig. 4(B)]: the pore of access to the buried Na^+^ site is open or closed in the free form, and Na^+^ binds to the open conformation with minimal changes of the structure.^57,58^ As a result of the pre-equilibrium, CS produces an apparent equilibrium dissociation constant

$$K_{d,\;\mathrm{app}}=\frac{E^{*}\left(\infty \right)+E\left(\infty \right)}{EX\left(\infty \right)}x=\frac{{k_{\mathrm{off}}(k}_{21}+k_{12})}{{k_{on}k}_{12}}=K_{d}\frac{k_{21}+k_{12}}{k_{12}}$$ (16)

that is always higher than the intrinsic $K_{d}$. As for the case of glucose binding to glucokinase, the value of $K_{d,\mathrm{app}}$ would be measured from titration of Na^+^ with thrombin at equilibrium, but would underestimate the affinity of the binding interaction to the E form by a factor of 9. When a binding interaction is interpreted in terms of CS, the affinity measured at equilibrium is the result of binding to a fraction of the total population of targets and always underestimates the affinity of binding to this population.

## IV. A PARADOX

**FIG. 5.**
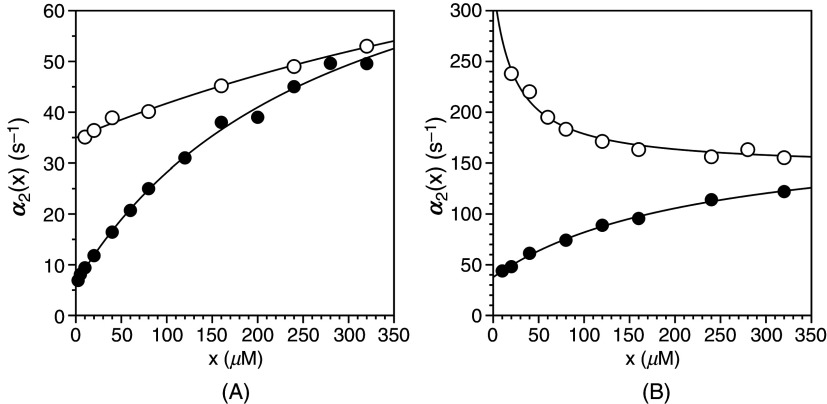
Rates of relaxation for FPR (filled circles) or FPK (open circles) binding to the thrombin mutant W215A^59^ under experimental conditions of: 50 mM Tris, 200 mM ChCl, 0.1% PEG8000, pH 8 at (A) 10 °C or (B) 25 °C. The data at 10 °C are consistent with IF under the rapid equilibrium approximation [Eq. (13a)] for both ligands. On the other hand, the data at 25 °C reveal a paradox where the thrombin mutant W215A binds FPR according to IF [Eq. (13a)] and FPK according to CS [Eq. (13b)] under the same experimental conditions. Continuous lines were drawn according to Eq. (14a) for IF and Eq. (14b) for CS. Adapted with permission from Ref. 59.

Although the rapid equilibrium approximation has attractive features and simplifies the mathematical expressions required to fit experimental data, it has potential limitations that may bias interpretation of the binding mechanism. Consider the case shown in Fig. 5(A) where the tripeptide FPR binds to the W215A mutant of thrombin according to a single saturable relaxation that increases hyperbolically with the ligand concentration x.^59^ The absence of a fast relaxation linked to the binding of FPR may be due to values of $k_{on}$ and $k_{\mathrm{off}}$ too fast to measure by stopped flow, or to silencing of the spectral signal linked to FPR binding due to mutation of W215, which is known to be a major fluorophore.^60^ When the data in Fig. 5(A) are interpreted according to IF under the rapid equilibrium approximation [Eq. (13a)], the relaxation measures the conformational transition of the thrombin-FPR intermediate to a final complex. The rate constants for this exchange are derived from the lower asymptote of the plot as $k_{32}$ = 6.3 ± 0.5 s^−1^ and the upper asymptote as the sum $k_{23}+k_{32}$= 90 ± 8 s^−1^, with $K_{d}$ = 280 ± 20 *μ*M. In this case, the value of $k_{32}$ is 13-fold higher than $k_{23},$ and the resulting value of $K_{d,\mathrm{app}}$ = 20 ± 2 *μ*M overestimates the affinity of the initial encounter by nearly 15-fold. When the same mutant interacts with the tripeptide H-D-Phe-Pro-Lys-p-nitroanilide (FPK), which differs from FPR for the presence of a Lys residue at the position occupied by Arg in FPR, a similar dependence of the relaxation is obtained with values of $k_{32}$ = 35 ± 3 s^−1^ and $k_{23}+k_{32}$ = 105 ± 9 s^−1^, with $K_{d}$ = 930 ± 80 *μ*M and $K_{d,\mathrm{app}}$= 310 ± 30 *μ*M. Interpretation according to IF under the rapid equilibrium approximation points to differences between the two ligands, with FPR binding more specifically because of a lower $K_{d,\mathrm{app}}$ that would be measured experimentally at equilibrium by fluorescence titrations or calorimetry. Importantly, the 15-fold difference in $K_{d,\mathrm{app}}$ caused by replacement of Arg with the less specific Lys at the P1 position^61^ of the ligand is due mainly to $k_{32}$, which measures the rate of conversion of E′X to EX and has nothing to do with the intrinsic binding affinity $K_{d}$. Hence, both ligands drive the EX→E′X transition with comparable rates, but FPR optimizes binding by better maintaining the complex in the E′X conformation. After the initial complex is formed, FPR induces a tighter fit toward a more stable final complex than the cognate ligand FPK.

Using IF for the analysis of FPR and FPK binding to the active site of the thrombin mutant W215A produces a compelling interpretation of the underlying mechanism and points to a strategy to optimize the affinity further. The pharmacology literature is full of similar interpretations that have informed the design of better lead compounds.^2,28,42,51^ However, when the binding interaction of FPR and FPK with the thrombin mutant W215A is studied at a higher temperature (25 °C), a different scenario emerges [Fig. 5(B)]. FPR binds according to IF as seen at 10 °C [Fig. 5(A)], with values of $k_{32}$ = 38 ± 3 s^−1^, $k_{23}+k_{32}$ = 180 ± 10 s^−1^, $K_{d}$ = 230 ± 20 *μ*M and $K_{d,\mathrm{app}}$ = 49 ± 5 *μ*M. The higher values of the rate constants $k_{23}$ and $k_{32}$ are expected at higher temperature, and so are the lower affinities $K_{d}$ and $K_{d,\mathrm{app}}$ due to a binding interaction that is enthalpically driven. On the other hand, binding of FPK is no longer consistent with IF but with CS, and the associated relaxation decreases from a value of $k_{12}+k_{21}$= 330 ± 30 s^−1^ to $k_{12}$ = 150 ± 10 s^−1^, with $K_{d}$ = 23 ± 2 *μ*M and $K_{d,\mathrm{app}}$ = 51 ± 5 *μ*M. How can the same protein behave according to two irreducible mechanisms like IF and CS under identical solution conditions? How can $E^{*}$ and E preexist in equilibrium for FPK but not FPR? The value of the saturable relaxation at x = 0 gives the rate constant $k_{32}$ for IF and the sum $k_{12}+k_{21}$ for CS. The former measures the rate for the E′X→EX transition, i.e., a property of the complex that may change with different ligands, as seen in Fig. 5(A). The latter measures the rate for the $E^{*}⇄\;E$ transitions to reach equilibrium, i.e., a property of the free macromolecule that must be independent of the ligand used. The data in Fig. 5(B) are inconsistent with IF because FPK clearly obeys CS, but they are also inconsistent with CS because FPR does not. A scenario similar to that shown in Figs. 5(A) and 5(B) for FPR and FPK binding to the W215A mutant of thrombin has been observed in P-type ATPases when using metal-fluoride complexes to induce E2P-like states with the aim of studying the events that occur during E2P hydrolysis.^62^ Although binding of BeF_x_ produces an increase in fluorescence analogous to Pi, it causes an increase in the value of α(x) with x according to IF, whereas Pi induces a decrease according to CS. Again, how can the P-type ATPase behave according to IF and CS under identical solution conditions? The paradoxical divergence in the kinetic behavior of a macromolecule toward two distinct ligands under identical solution conditions is resolved once we abandon the rapid equilibrium approximation.

## V. IF IN THE GENERAL CASE

How different is the behavior of the system in the general case, i.e., without the rapid equilibrium approximation? This question was first addressed by simulations using the complex kinetics of MANT-ADP binding to DnaC^63^ and then in general mathematical terms for IF [Eq. (11)] and CS [Eq. (12)].^64^ Consider the properties of IF under the general assumptions used for the lock and key mechanism [Eq. (9)] and with the ligand in excess over the macromolecule. Because the system is closed, only two of the three macromolecular species in Eq. (11) are independent and the time evolution of the system is

$$\left(\begin{array}{c} dE/dt\\ dEX/dt\\ dE\prime X/dt \end{array}\right)=\left(\begin{array}{ccc} {-k}_{on}x & k_{\mathrm{off}} & 0\\ k_{on}x & -k_{\mathrm{off}}-k_{23} & k_{32}\\ 0 & k_{23} & {-k}_{32} \end{array}\right)\left(\begin{array}{c} E\\ EX\\ E\prime X \end{array}\right)$$ (17)

or

$$E\left(t\right)=c_{0}A_{10}+c_{1}A_{11}e^{-\alpha_{1}\left(x\right)t}+c_{2}A_{12}e^{-\alpha_{2}\left(x\right)t}=E\left(\infty \right)+c_{1}A_{11}e^{-\alpha_{1}\left(x\right)t}+c_{2}A_{12}e^{-\alpha_{2}\left(x\right)t}$$ (18a)

$$EX\left(t\right)=c_{0}A_{20}+c_{1}A_{21}e^{-\alpha_{1}\left(x\right)t}+c_{2}A_{22}e^{-\alpha_{2}\left(x\right)t}=EX\left(\infty \right)+c_{1}A_{21}e^{-\alpha_{1}\left(x\right)t}+c_{2}A_{22}e^{-\alpha_{2}\left(x\right)t}$$ (18b)

$$E^{\prime }X\left(t\right)=c_{0}A_{30}+c_{1}A_{31}e^{-\alpha_{1}\left(x\right)t}+c_{2}A_{32}e^{-\alpha_{2}\left(x\right)t}=E\prime X\left(\infty \right)+c_{1}A_{31}e^{-\alpha_{1}\left(x\right)t}+c_{2}A_{32}e^{-\alpha_{2}\left(x\right)t}.$$ (18c)

Here the *A*'s are elements of the eigenvectors of the matrix in Eq. (17); the *c*'s are arbitrary constants that satisfy the initial conditions $E\left(0\right)$, $EX\left(0\right),$ and $E\prime X\left(0\right)$; and $E\left(\infty \right)$, $EX\left(\infty \right),$ and $E\prime X\left(\infty \right)$ are the values of $E\left(t\right)$, $EX\left(t\right),$ and $E\prime X\left(t\right)$ at t=∞. The α's are the rates of approach to equilibrium defined as the two non-zero eigenvalues, with changed sign, $\alpha_{1}(x)$ and $\alpha_{2}(x)$ of the matrix in Eq. (17), i.e.,

$$\alpha_{1}\left(x\right)=\frac{1}{2}\{k_{on}x+k_{\mathrm{off}}+k_{23}+k_{32}+\sqrt{{\left(k_{on}x+k_{\mathrm{off}}-k_{23}-k_{32}\right)}^{2}+4{k_{\mathrm{off}}k}_{23}}\}$$ (19a)

$$\alpha_{2}\left(x\right)=\frac{1}{2}\{k_{on}x+k_{\mathrm{off}}+k_{23}+k_{32}-\sqrt{{\left(k_{on}x+k_{\mathrm{off}}-k_{23}-k_{32}\right)}^{2}+4{k_{\mathrm{off}}k}_{23}}\}.$$ (19b)

The sums $k_{on}x+k_{\mathrm{off}}$ and $k_{23}+k_{32}$ measure the rates at which the exchanges E+X ⇄  EX and EX ⇄  E′X reach equilibrium. They make a symmetric contribution to Eqs. 19(a) and 19(b), i.e., they can be swapped without affecting the behavior of the system. The term ${k_{\mathrm{off}}k}_{23}$ breaks the symmetry and depends on the two rate constants that deplete the EX intermediate in Eq. (11). The functional behavior of the IF mechanism in the general case depends not on the relative rates at which binding E+X ⇄  EX and conformational transitions EX ⇄  E′X reach equilibrium. Rather, it depends on how fast the EX intermediate disappears as a result of ligand dissociation ( $k_{\mathrm{off}}$) and conversion to the more stable complex ( $k_{23}$). The limiting values of the relaxations in Eqs. (19a) and (19b) are easily calculated as

$$\alpha_{1}(0)=\frac{1}{2}\left\{k_{\mathrm{off}}+k_{23}+k_{32}+\sqrt{{\left(k_{\mathrm{off}}-k_{23}-k_{32}\right)}^{2}+4{k_{\mathrm{off}}k}_{23}}\right\}$$ (20a)

$$\alpha_{1}(\infty )\approx k_{on}x$$ (20b)

$$\alpha_{2}(0)=\frac{1}{2}\left\{k_{\mathrm{off}}+k_{23}+k_{32}-\sqrt{{\left(k_{\mathrm{off}}-k_{23}-k_{32}\right)}^{2}+4{k_{\mathrm{off}}k}_{23}}\right\}$$ (20c)

$$\alpha_{2}\left(\infty \right)=k_{23}+k_{32}.$$ (20d)

The fast relaxation, $\alpha_{1}(x)$, always increases with x and eventually grows linearly as x→∞. In this limit [Eq. (20b)], the relaxation becomes indistinguishable from that describing the simple lock-and-key mechanism because it is dominated by the binding interaction. The slow relaxation, $\alpha_{2}(x)$, also increases with x, but saturates at a value $\alpha_{2}\left(\infty \right)=k_{23}+k_{32}$ that reflects the rate at which the EX ⇄  E′X exchange reaches equilibrium [Eq. (20d)]. This limiting value is identical to that seen in the case of IF under the rapid equilibrium approximation [Eq. (14a)]. Under saturating conditions of ligand, the system partitions between its main processes E+X→EX, reflecting formation of the complex, and EX ⇄  E′X, reflecting the conformational transition once the complex is formed. This asymptotic value depends on properties of the complex and is expected to be different for different ligands. In the absence of ligand, the two transitions make overlapping contributions to the kinetics that depend on all rate constants in the kinetic scheme [Eqs. (20a) and (20c)], except $k_{on}$. Again, these values are expected to be influenced by the particular ligand under consideration. We also note that the difference between the lower limit of the fast relaxation and the upper limit of the slow relaxation defines an important quantity ε such that

$$\alpha_{1}\left(0\right)-\alpha_{2}\left(\infty \right)=\frac{1}{2}\{k_{\mathrm{off}}-k_{23}-k_{32}+\sqrt{{\left(k_{\mathrm{off}}-k_{23}-k_{32}\right)}^{2}+4{k_{\mathrm{off}}k}_{23}}\}=\varepsilon$$ (21a)

$$\alpha_{2}\left(0\right)+\varepsilon =k_{\mathrm{off}}.$$ (21b)

When the mechanism of binding obeys IF, the value of ε can be derived directly by inspection of the plot of the two relaxations. Once the value of ε is known, the rate constant $k_{\mathrm{off}}$ is also derived by inspection as the sum of the lower limit of the slow relaxation and ε [Eq. (21b)]. The value of ε ranges from 0 to $k_{\mathrm{off}}$, and its importance will become apparent in Sec. VII.

Both relaxations in the IF mechanism feature a monotonic increase with the ligand concentration x, as shown by the derivative

$$\frac{d\alpha_{1,2}(x)}{dx}=\frac{k_{on}}{2}\left(1\pm \frac{k_{on}x+k_{\mathrm{off}}-k_{23}-k_{32}}{\sqrt{{\left(k_{on}x+k_{\mathrm{off}}-k_{23}-k_{32}\right)}^{2}+4{k_{\mathrm{off}}k}_{23}}}\right)>0.$$ (22)

Therefore, IF cannot account for the kinetic profile shown in Fig. 4(A), even in the general case. It is instructive to show how Eqs. (19a) and (19b) change in the rapid equilibrium approximation, which is obtained by assuming that $k_{on}x+k_{\mathrm{off}}\gg k_{23}+k_{32}$ for all values of x. Simple algebra yields the expressions

$$\alpha_{1}(x)=k_{on}x+k_{\mathrm{off}}$$ (23a)

$$\alpha_{2}\left(x\right)=\underset{k_{\mathrm{on}}x+k_{\mathrm{off}\to \infty }}{\lim}\frac{{\left(k_{on}x+k_{\mathrm{off}}+k_{23}+k_{32}\right)}^{2}-{\left(k_{on}x+k_{\mathrm{off}}-k_{23}-k_{32}\right)}^{2}-4{k_{\mathrm{off}}k}_{23}}{k_{on}x+k_{\mathrm{off}}+k_{23}+k_{32}+\sqrt{{\left(k_{on}x+k_{\mathrm{off}}-k_{23}-k_{32}\right)}^{2}+4{k_{\mathrm{off}}k}_{23}}}=k_{23}\frac{x}{K_{d}+x}+k_{32}=k_{23}f+k_{32}.$$ (23b)

Separation of the time scales for binding ( $k_{on}x+k_{\mathrm{off}}$) and conformational change ( $k_{23}+k_{32}$) produces the expected expressions: the fast relaxation is identical to the lock-and-key model [Eq. (10)], and the slow relaxation is identical to Eq (14a). The lower limit of the fast relaxation separates widely from the asymptotic upper limit of the slow relaxation and the value of ε increases. Hence, a necessary condition for invoking the rapid equilibrium approximation is that the two relaxations be widely separated, as seen for the case of glucose binding to glucokinase, shown in Fig. 3(A). However, the condition is not sufficient, as we will see in Secs. VI and VII. In the general case, Eq. (11) depends on four independent parameters that can be resolved only when both $\alpha_{1}(x)$ and $\alpha_{2}(x)$ are measured experimentally. If only the slow relaxation is accessible to experimental measurements as a hyperbolic increase as a function of x, then none of the four independent values of the IF mechanism can be resolved unequivocally from Eqs. (20c) and (20d). Parameters estimated through the simplified Eq. (23b) under the rapid equilibrium approximation may not give a correct interpretation of the underlying kinetic mechanism.

## VI. CS IN THE GENERAL CASE

The properties of the mechanism of CS [Eq. (12)] in the general case have been discussed only recently,^64^ notwithstanding their mathematical simplicity. Under the assumption that the system is closed and the ligand is in large excess over the macromolecule, only two of the three macromolecular species in Eq. (12) are independent, and the time evolution of the system is

$$\left(\begin{array}{c} dE^{*}/dt\\ dE/dt\\ dEX/dt \end{array}\right)=\left(\begin{array}{ccc} {-k}_{12} & k_{21} & 0\\ k_{12} & -k_{on}x-k_{21} & k_{\mathrm{off}}\\ 0 & k_{on}x & {-k}_{\mathrm{off}} \end{array}\right)\left(\begin{array}{c} E^{*}\\ E\\ EX \end{array}\right)$$ (24)

or

$$E^{*}\left(t\right)=c_{0}A_{10}+c_{1}A_{11}e^{-\alpha_{1}\left(x\right)t}+c_{2}A_{12}e^{-\alpha_{2}\left(x\right)t}=E^{*}\left(\infty \right)+c_{1}A_{11}e^{-\alpha_{1}\left(x\right)t}+c_{2}A_{12}e^{-\alpha_{2}\left(x\right)t}$$ (25a)

$$E\left(t\right)=c_{0}A_{20}+c_{1}A_{21}e^{-\alpha_{1}\left(x\right)t}+c_{2}A_{22}e^{-\alpha_{2}\left(x\right)t}=E\left(\infty \right)+c_{1}A_{21}e^{-\alpha_{1}\left(x\right)t}+c_{2}A_{22}e^{-\alpha_{2}\left(x\right)t}$$ (25b)

$$EX\left(t\right)=c_{0}A_{30}+c_{1}A_{31}e^{-\alpha_{1}\left(x\right)t}+c_{2}A_{32}e^{-\alpha_{2}\left(x\right)t}=EX\left(\infty \right)+c_{1}A_{31}e^{-\alpha_{1}\left(x\right)t}+c_{2}A_{32}e^{-\alpha_{2}\left(x\right)t}.$$ (25c)

The parameters in Eqs. (25a)–(25c) are analogous to those for IF in Eqs. (18a)–(18c), and the relevant expressions for the relaxations α's are

$$\alpha_{1}\left(x\right)=\frac{1}{2}\{k_{on}x+k_{\mathrm{off}}+k_{12}+k_{21}+\sqrt{{\left(k_{on}x+k_{\mathrm{off}}-k_{12}-k_{21}\right)}^{2}+4k_{21}k_{on}x}\}$$ (26a)

$$\alpha_{2}\left(x\right)=\frac{1}{2}\{k_{on}x+k_{\mathrm{off}}+k_{12}+k_{21}-\sqrt{{\left(k_{on}x+k_{\mathrm{off}}-k_{12}-k_{21}\right)}^{2}+4k_{21}k_{on}x}\}.$$ (26b)

The similarity of Eqs. (26a) and (26b) to Eqs. (19a) and (19b) is evident. There is a symmetric contribution from the sums of rate constants defining the relaxation to equilibrium of the two separate processes $E^{*}\;⇄\;\;E$, reflecting the conformational transition, and E+X ⇄  EX, reflecting binding, whereas the term $k_{21}k_{on}x$ makes an asymmetric contribution that depends on rate constants that deplete the intermediate species E in the reaction scheme [Eq. (12)]. Notably, this term is a constant for IF ( ${k_{\mathrm{off}}k}_{23}$), but a function of the ligand concentration x for CS ( $k_{21}k_{on}x$), which presages important differences in the kinetic properties of CS compared to IF. The expectation that CS may be mathematically and functionally more versatile than IF is at odds with the documented paucity of systems that behave according to CS based on the rapid equilibrium approximation.^28,33^ Yet, the unique features of CS are immediately evident once we consider the limiting values of the two relaxations, i.e.,

$$\alpha_{1}\left(0\right)={\mathrm{larger}\;of\;k}_{\mathrm{off}}\;or\;k_{12}+k_{21}$$ (27a)

$$\alpha_{1}(\infty )\approx k_{on}x$$ (27b)

$$\alpha_{2}(0)={\mathrm{smaller}\;of\;k}_{\mathrm{off}}\;or\;k_{12}+k_{21}$$ (27c)

$$\alpha_{2}(\infty )=k_{12}.$$ (27d)

The upper limit of the fast relaxation, $\alpha_{1}(\infty )$, grows linearly as x→∞, as seen for IF [Eq. (20b)]. The analogous limit for the slow relaxation, $\alpha_{2}(\infty )$, saturates at a value equal to $k_{12},$ defining the rate constant for the $E^{*}\to E$ transition, which is a property of the free macromolecule. This is somewhat counterintuitive, because the behavior of the system under saturating concentrations of ligand is expected to depend on properties of the bound forms of the macromolecule. Indeed, the value of $\alpha_{2}(\infty )$ for IF reflects properties of the complex [Eq. (20d)]. Furthermore, the value of $\alpha_{2}(\infty )$ for CS unequivocally defines one of the rate constants in the kinetic mechanism [Eq. (12)], which can be derived from inspection of the relaxation plot, without the need for data analysis. The lower limits of the two relaxations, $\alpha_{1}(0)$ and $\alpha_{2}(0)$, can only be assigned unequivocally once the relative values of $k_{\mathrm{off}}$ and $k_{12}+k_{21}$ are known. The larger value is assigned to the fast relaxation, and the smaller one to the slow relaxation. This is a consequence of the term $k_{21}k_{on}x$ in Eqs. (26a) and (26b), no longer contributing to the two relaxations when x→0. When $k_{\mathrm{off}}>k_{12}+k_{21}$, the fast relaxation has a lower limit $\alpha_{1}(0)$ that depends entirely on properties of the complex, and the slow relaxation has both limits, $\alpha_{2}(0)$ and $\alpha_{2}\left(\infty \right)$, which depend entirely on properties of the free macromolecule. When $k_{\mathrm{off}}<k_{12}+k_{21}$, the lower limit of the fast relaxation no longer reflects properties of the complex but rather of the free macromolecule, whereas the value of $\alpha_{2}(0)$ reflects properties of the complex and no longer of the free macromolecule. In this case, the behavior of the slow relaxation reflects properties of the complex when x→0 and the macromolecule is mostly in the free form, but it reflects properties of the free macromolecule when x→∞ and the macromolecule is mostly in the bound form. The peculiar contribution of the term $k_{21}k_{on}x$ in Eqs. (26a) and (26b) becomes fully apparent when the dependence of each relaxation is studied as a function of the ligand concentration [Eqs. (27a)–(27d)].

The fast relaxation of CS is expected to increase as a function of x from the values of the limits in Eqs. (27a) and (27b). Indeed, the derivative

$$\frac{d\alpha_{1}(x)}{dx}=\frac{k_{on}}{2}\left(1+\frac{k_{on}x+k_{\mathrm{off}}{-k}_{12}+k_{21}}{\sqrt{{\left(k_{on}x+k_{\mathrm{off}}-k_{12}-k_{21}\right)}^{2}+4k_{21}k_{on}x}}\right)>0.$$ (28a)

A quite different scenario is observed for the slow relaxation, whose behavior depends on the sign of the expression $k_{\mathrm{off}}-k_{12}$. The derivative

$$\frac{d\alpha_{2}(x)}{dx}=\frac{k_{on}}{2}\left(1-\frac{k_{on}x+k_{\mathrm{off}}-k_{12}+k_{21}}{\sqrt{{\left(k_{on}x+k_{\mathrm{off}}-k_{12}-k_{21}\right)}^{2}+4k_{21}k_{on}x}}\right)<0\;\text{for}\;k_{\mathrm{off}}>k_{12}$$ (28b)

$$\frac{d\alpha_{2}(x)}{dx}=\frac{k_{on}}{2}\left(1-\frac{k_{on}x+k_{\mathrm{off}}-k_{12}+k_{21}}{\sqrt{{\left(k_{on}x+k_{\mathrm{off}}-k_{12}-k_{21}\right)}^{2}+4k_{21}k_{on}x}}\right)>0\;\text{for}\;k_{\mathrm{off}}<k_{12}$$ (28c)

$$\frac{d\alpha_{2}(x)}{dx}=\frac{k_{on}}{2}\left(1-\frac{k_{on}x+k_{\mathrm{off}}-k_{12}+k_{21}}{\sqrt{{\left(k_{on}x+k_{\mathrm{off}}-k_{12}-k_{21}\right)}^{2}+4k_{21}k_{on}x}}\right)=0\;\text{for}\;k_{\mathrm{off}}=k_{12}.$$ (28d)

**FIG. 6.**
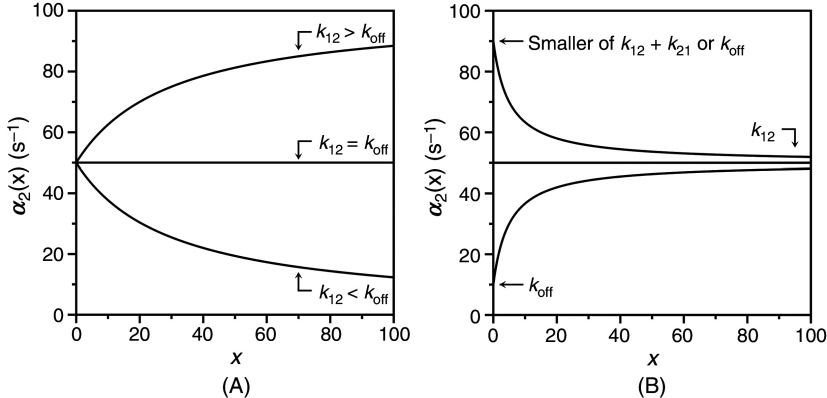
Kinetic behavior of the slow relaxation of CS in the general case [Eq. (26b)] showing the effect of changing $k_{12}$ while keeping $k_{\mathrm{off}}$ (A) constant, or (B) vice versa. The simulations in panel (A) provide an explanation for the data on FPR binding to prothrombin and its derivatives [Fig 7(A)]. The simulations in panel (B) resolve the paradox discussed in Sec. IV about the binding of FPR and FPK to the thrombin mutant W215A (Fig 5).

We have already established [Eq. (27c)] that the smaller value between $k_{\mathrm{off}}$ and $k_{12}+k_{21}$ is assigned to $\alpha_{2}\left(0\right),$ and this introduces variability in the relative values of $\alpha_{2}(0)$ and $\alpha_{2}\left(\infty \right)$. The relaxation $\alpha_{2}(x)$ always decreases with x when $\alpha_{2}\left(0\right)=k_{12}+k_{21}$, but it may also decrease when $k_{\mathrm{off}}>k_{12}$. In addition, the relaxation increases with x whenever $k_{\mathrm{off}}<k_{12}$ and is independent of x in the special case when $k_{\mathrm{off}}=k_{12}$. The different behaviors of the slow relaxation of CS in the general case are illustrated in Fig. 6, where the value of $k_{12}$ is changed while keeping $k_{\mathrm{off}}$ constant [Fig. 6(A)], or vice versa [Fig. 6(B)]. The examples in Fig. 6(B) offer a possible solution to the paradox presented in Fig. 5. The data at 10 °C [Fig. 5(A)] show the two ligands FPR and FPK obeying a single relaxation that increases hyperbolically with x and converging toward a similar upper asymptote. This kinetic profile would be interpreted with IF under the rapid equilibrium approximation, but is also consistent with CS in the general case. The value of $\alpha_{2}\left(\infty \right)=k_{12}$ [Eq. (27d)] for CS reflects a property of the free macromolecule and should be independent of the ligand used, as indeed observed for FPR and FPK [Fig. 5(A)], and for BeF_x_ and Pi binding to the P-type ATPase.^62^ The difference between the ligands in the lower asymptote is explained by the difference in the value of $k_{\mathrm{off}}<k_{12}$, i.e., the two ligands bind to the thrombin mutant W215A with the same mechanism of CS but with different values of $k_{\mathrm{off}}$. The conclusion is supported by the data collected at 25 °C [Fig. 5(B)] that again converge toward the same value of $\alpha_{2}\left(\infty \right)=k_{12},$ but differ sharply in the value of $k_{\mathrm{off}}$. As this value grows above the value of $k_{12}$ that is independent of the ligand, the condition $k_{\mathrm{off}}>k_{12}$ takes hold and inverts the dependence of $\alpha_{2}\left(x\right)$ on x from a hyperbolic *increase* for FPR to a hyperbolic *decrease* for FPK. The same conclusion applies to the case of BeF_x_ and Pi binding to the P-type ATPase: both ligands obey CS but differ in the value of $k_{\mathrm{off}}$ that is significantly higher in the case of Pi.^62^ The data in Fig. 5 and the simulations in Fig. 6 demonstrate the importance of interrogating the kinetic behavior of the system with different ligands and under different experimental conditions. Data limited to FPR, even at different temperatures, would have supported IF under the rapid equilibrium approximation as a mechanism of binding to the thrombin mutant W215A. The same conclusion would have been drawn from analysis of FPK binding at 10 °C or analysis of BF_x_ binding to the P-type ATPase.^62^

**FIG. 7.**
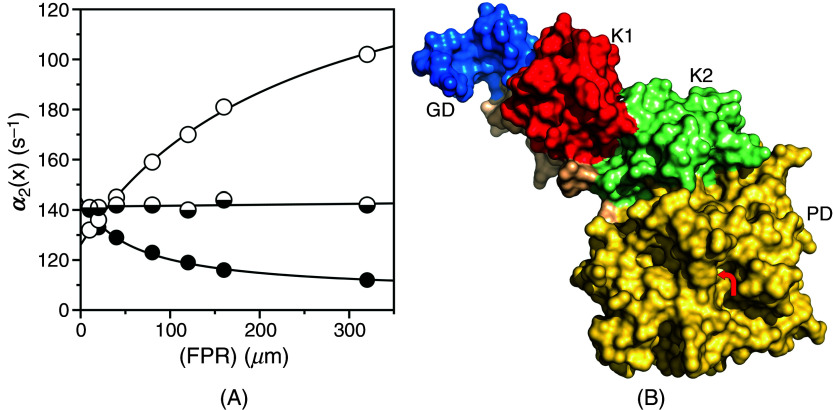
(A) Rapid kinetics of FPR binding to prothrombin (closed circles), prethrombin-1 (mixed circles), and prethrombin-2 (open circles)^65^ showing how the slow relaxation for the three proteins is consistent with CS in the general case [Eq. (12)] and the simulations in Fig. 6(A). The kinetic profiles of prothrombin, prethrombin-1, and prethrombin-2 differ markedly, especially in the value of $k_{12}$. Experimental conditions are: 400 mM ChCl, 50 mM Tris, 0.1% PEG8000, pH 8.0, at 15 °C. (B) Crystal structure of prothrombin^70^ that reveals the multi domain architecture of the protein composed of the Gla domain (GD, blue), kringle-1 (K1, red), kringle-2 (K2, green), and protease domain (PD, gold) containing the binding site for the ligand FPR (arrow). Removal of the Gla domain and kringle-1 generates the intermediate prethrombin-1,^66^ and further deletion of kringle-2 frees the protease domain as prethrombin-2.^67^

The relevance of CS in the general case is further documented by the simulations shown in Fig. 6(A) that bear direct relevance to ligand binding to the active site of the clotting factor prothrombin^65^ and its two smaller derivatives, prethrombin-1^66^ and prethrombin-2.^67^ Prothrombin is composed of 579 residues and has a modular assembly [Fig. 7(B)] that comprises the Gla-domain, two kringles, and the protease domain housing the active site.^68^ Binding of the ligand FPR to prothrombin produces a single relaxation, decreasing hyperbolically with the ligand concentration that proves unequivocally the validity of CS as a mechanism of binding [Eq. (28a)] and rules out IF [Fig. 7(A)]. The limiting values in the plot are $\alpha_{2}^{\;}\left(0\right)$ = 45 ± 5 s^−1^, defining the smaller between $k_{\mathrm{off}}$ or the sum $k_{12}+k_{21}$, and $\alpha_{2}^{\;}\left(\infty \right)$ = 6.9 ± 0.3 s^−1^ defining the value of $k_{12}$. Cleavage at residue R155 between the two kringles of prothrombin removes the Gla domain and kringle-1 and generates prethrombin-1 [Fig. 7(B)]. FPR binding to prethrombin-1 features a single relaxation independent of ligand concentration [Fig. 7(A)] that again proves unequivocally the validity of CS [Eq. (28c)] and leads to the conclusion that $\alpha_{2}^{\;}\left(0\right)=\alpha_{2}^{\;}\left(\infty \right)$, or else $k_{\mathrm{off}}^{\;}=k_{12}$= 42 ± 2 s^−1^. The value compares well to $\alpha_{2}^{\;}\left(0\right)$ = 45 ± 5 s^−1^ measured for prothrombin and suggests that this limit in the slow relaxation of prothrombin likely measures $k_{\mathrm{off}}$ and that this rate constant does not change significantly upon removal of the Gla domain and kringle-1. What changes in the transition from prothrombin to prethrombin-1 is the value of $k_{12}$, measuring the rate of opening of the active site in the $E^{*}\to E$ transition that increases significantly in prethrombin-1. Hence, removal of the Gla domain and kringle-1 affects the intrinsic $E^{*}\;⇌\;E$ equilibrium, controlling ligand binding to the active site in the protease domain located > 50 Å away. Removal of kringle-2 from prethrombin-1 further reduces the size of the protein and generates prethrombin-2 [Fig. 7(B)]. In this case, FPR binding produces a single relaxation that increases hyperbolically with the ligand concentration [Fig. 7(A)]. It is possible that the drastic structural perturbation produced by transitioning from prothrombin to prethrombin-2 causes the molecular mechanism of recognition of FPR to change from CS to IF. However, it is reasonable to assume that the mechanism of FPR binding does not change and that the data for prethrombin-2 can also be interpreted with CS to give $\alpha_{2}^{\;}\left(0\right)=k_{\mathrm{off}}$ = 27 ± 2 s^−1^ and $\alpha_{2}^{\;}\left(\infty \right)=k_{12}$ = 170 ± 10 s^−1^. The value of $k_{\mathrm{off}}$ is similar to that measured for prothrombin and prethrombin-1 and the value of $k_{12},$ measuring the rate of opening of the active site in the $E^{*}\to E$ increases even further compared to prethrombin-1. Hence, FPR binding to prothrombin and its smaller derivatives can be explained in terms of CS, with a value of $k_{\mathrm{off}}$ that remains fairly constant and a value of $k_{12}$ that becomes progressively faster as the Gla domain and kringles are removed from the structure. This interpretation is consistent with the conformational plasticity of prothrombin documented by X-ray structural biology^69,70^ as the driving force producing long-range perturbation of the active site from the auxiliary Gla domain and kringles.

The versatility of CS and Eq. (26b) is lost under the rapid equilibrium approximation, which is obtained under the assumption $k_{on}x+k_{\mathrm{off}}\gg k_{12}+k_{21}$. Simple algebra yields the expressions

$$\alpha_{1}(x)=k_{on}x+k_{\mathrm{off}}$$ (29a)

$$\alpha_{2}\left(x\right)=\underset{k_{\mathrm{on}}x+k_{\mathrm{off}\to \infty }}{\lim}\frac{{\left(k_{on}x+k_{\mathrm{off}}+k_{12}+k_{21}\right)}^{2}-{\left(k_{on}x+k_{\mathrm{off}}-k_{12}-k_{21}\right)}^{2}-4{k_{21}k}_{on}x}{k_{on}x+k_{\mathrm{off}}+k_{12}+k_{21}+\sqrt{{\left(k_{on}x+k_{\mathrm{off}}-k_{23}-k_{32}\right)}^{2}+4{k_{\mathrm{off}}k}_{23}}}=k_{12}+k_{21}\frac{K_{d}}{K_{d}+x}=k_{12}+k_{21}(1-f).$$ (29b)

The fast relaxation becomes the familiar expression for the lock-and-key model [Eq. (10)] and the slow relaxation becomes identical to Eq. (14b), which always decreases as a function of x. The important conclusion from this analysis is that a simple distinction between IF and CS is only possible under the rapid equilibrium approximation. In the general case, CS produces kinetics that are incompatible with IF because $\alpha_{2}\left(\infty \right)<\alpha_{2}(0)$, thereby explaining experimental data that can only be interpreted with CS. In addition, CS is consistent with experimental data where $\alpha_{2}\left(\infty \right)>\alpha_{2}(0)$ that might be considered unequivocal proof of IF. Finally, CS also accounts for the case of a relaxation that is independent of the ligand concentration, i.e., ${\alpha_{2}\left(x\right)=\alpha }_{2}\left(\infty \right)=\alpha_{2}(0)$, that finds no explanation in terms of either IF or CS under the rapid equilibrium approximation.^71^ The properties of CS in the general case [Eqs. (26a) and (26b)] have significant practical implications for the analysis of experimental data. The fast relaxation $\alpha_{1}(x)$ has a behavior compatible with both IF and CS, and eventually increases linearly with x because it monitors the effect of ligand binding. The slow relaxation, on the other hand, saturates at high x to values that convey information on the conformational properties of the complex for IF or the free species for CS. When the slow relaxation decreases hyperbolically with x, the system unequivocally obeys CS, and IF is necessarily ruled out. The same conclusion applies to the case of a slow relaxation being independent of x. On the other hand, when the slow relaxation increases hyperbolically with x, the system may obey IF or CS, and neither mechanism is necessarily ruled out. CS is always sufficient as an explanation for the mechanism of ligand binding and is also necessary when a saturable relaxation decreases hyperbolically with x. In contrast, IF is never necessary as an explanation for the mechanism of ligand binding and is only sufficient when a saturable relaxation increases hyperbolically with x.

The greater versatility of CS compared to IF has direct relevance to enzyme kinetics. Formation of a catalytically active complex according to CS may show a change in the rate-limiting step from trapping the E intermediate at low x to the rate $k_{12}$ for the $E^{*}\to E$ transition at high x. The richer functional repertoire of CS and its potential overlap with IF was first uncovered using simulations of a more complex kinetic scheme involving both IF and CS.^63^ Surprisingly, however, the full mathematical complexity of the simple schemes for IF [Eq. (11)] and CS [Eq. (12)] reported above has been documented only recently.^64,72^

## VII. IF AS A MATHEMATICAL SPECIAL CASE OF CS

In its general form, CS is not only more versatile than IF but it includes IF as a mathematical special case. The entire repertoire of kinetic properties of IF can be recapitulated by CS, but the reverse is not true. Any experimental data set consistent with IF can be interpreted with identical mathematical accuracy with CS. This result is unexpected given that IF and CS have long been assumed to be mutually exclusive mechanisms and mathematically irreducible.^4,5,22,23,28,33^ The result is also counterintuitive because IF and CS are two-step reactions where binding and conformational changes are linked in sequence, but in reverse order. Why should binding preceded by a conformational change be functionally more versatile than the reverse sequence of events? And why should it include the reverse order as a special case? Functional complexity seems to depend on where conformational transitions take place in the kinetic mechanism, which generates hierarchy within the same topology of the reaction scheme. The relaxations for the IF and CS in Eqs. (19a) and (19b) and Eqs. (26a) and (26b) can be rewritten as

$$\alpha_{1,2}^{IF}\left(x\right)=\frac{1}{2}\{k_{23}+k_{32}+k_{on}^{IF}x+k_{\mathrm{off}}^{IF}\pm \sqrt{{\left(k_{on}^{IF}x+k_{\mathrm{off}}^{IF}-k_{23}-k_{32}\right)}^{2}+4k_{23}k_{\mathrm{off}}^{IF}}\}$$ (30a)

$$\alpha_{1,2}^{CS}\left(x\right)=\frac{1}{2}\{k_{12}+k_{21}+k_{on}^{CS}x+k_{\mathrm{off}}^{CS}\pm \sqrt{{\left(k_{on}^{CS}x+k_{\mathrm{off}}^{CS}-k_{12}-k_{21}\right)}^{2}+4k_{21}k_{on}^{CS}x}\}$$ (30b)

to better identify rate constants that apply to each mechanism. Because IF only produces relaxations that increase monotonically with x [Eq. (22)], we will prove that this mechanism is a special case of CS under the condition $k_{\mathrm{off}}^{CS}<k_{12}$ that also produces relaxations that increase monotonically with x [Eqs. (26b) and (28c)]. Simple algebra shows that Eq. (30a) of IF is mathematically identical to Eq. (30b) of CS for any value of x under the following transformations:^59^

$$k_{on}^{CS}=k_{on}^{IF}$$ (31a)

$$k_{12}=k_{23}+k_{32}$$ (31b)

$$k_{21}=\varepsilon$$ (31c)

$$k_{\mathrm{off}}^{CS}=k_{\mathrm{off}}^{IF}-\varepsilon ,$$ (31d)

where ε was defined in Eq. (21a). The first condition [Eq. (31a)] is a consequence of the fast relaxation increasing linearly as $k_{on}x$ for x→∞ in Eqs. (20b) and (27b). The second condition [Eq. (31b)] equates the limiting values of the slow relaxation for x→∞ in Eqs. (20d) and (27d). The third condition [Eq. (31c)] equates the values of the fast relaxation for x→0 in Eqs. (20a) and (27a), i.e.,

$$k_{12}+k_{21}=\frac{1}{2}\{k_{\mathrm{off}}+k_{23}+k_{32}+\sqrt{{\left(k_{\mathrm{off}}-k_{23}-k_{32}\right)}^{2}+4{k_{\mathrm{off}}k}_{23}}\},$$ (32)

and uses the definition of ε in Eq. (21a). Alternatively, Eq. (31c) equates the spacing between the lower limit of the fast relaxation and the upper limit of the slow relaxation which is given by ε in IF and by the difference $k_{12}+k_{21}-k_{12}=k_{21}$ in CS. The fourth condition [Eq. (31d)] equates Eqs. (20c) and (27c), using again the definition of ε in Eq. (21a). The four conditions in Eqs. (31a)–(31d) make the two expressions in Eqs. (30a) and (30b) mathematically identical for all values of the independent variable x. Therefore, experimental data consistent with IF [Eq. (30a)] can be fit with identical mathematical accuracy with CS [Eq. (30b)] using the following equivalence:

$$E\begin{array}{c} k_{on}x\\ \;⇄\;\\ k_{\mathrm{off}} \end{array}EX\begin{array}{c} k_{23}\\ \;⇄\;\\ k_{32} \end{array}E\prime X=E^{*}\begin{array}{c} k_{23}+k_{32}\\ \;⇄\;\\ \varepsilon \end{array}E\begin{array}{c} k_{on}x\\ \;⇄\;\\ k_{\mathrm{off}}-\varepsilon \end{array}EX.$$ (33)

To illustrate the importance of Eq. (33), we revisit the binding of glucose to glucokinase dealt with in Fig. 3(A). The two relaxations determined experimentally^54^ were originally interpreted in terms of IF under the rapid equilibrium approximation. Using IF in the general case offers a global fit of the data with best-fit parameter values: $k_{on}$ = 540 ± 20 M^−1^s^−1^, $k_{\mathrm{off}}$ = 7.5 ± 0.2 s^−1^, $k_{23}$ = 0.44 ± 0.02 s^−1^, $k_{32}$ = 0.36 ± 0.01 s^−1^. The mathematically equivalent interpretation in terms of CS generates best-fit parameter values: $k_{on}$= 540 ± 20 M^−1^s^−1^, $k_{\mathrm{off}}$ = 0.34 ± 0.01 s^−1^, $k_{12}$ = 0.80 ± 0.02 s^−1^, $k_{21}$ = 7.2 ± 0.2 s^−1^. Application of Eq. (33) yields the equivalence

$$E\begin{array}{c} 540\;{M^{-1}s}^{-1}x\;\\ \;⇄\;\\ 7.5\;s^{-1}\; \end{array}EX\begin{array}{c} 0.44\;s^{-1}\\ \;⇄\;\\ 0.36\;s^{-1} \end{array}E\prime X=E^{*}\begin{array}{c} 0.80\;s^{-1}\\ \;⇄\;\\ 7.2\;s^{-1} \end{array}E\begin{array}{c} 540\;{M^{-1}s}^{-1}x\\ \;⇄\;\\ 0.34\;s^{-1} \end{array}EX.$$ (34)

Binding of glucose to glucokinase can be interpreted with IF as a relatively low affinity intrinsic binding step ( $K_{d}$ = 14 mM) followed by a slight twofold increase in affinity due to a conformational transition of the complex equilibrating at a rate $k_{23}+k_{32}$ = 0.80 s^−1^. Combination of binding and conformational transitions results in a value of $K_{d,\mathrm{app}}$ = 6.3 mM from Eq. (15), which would be obtained from equilibrium measurements. The value slightly overestimates the intrinsic binding affinity of glucose to glucokinase. Alternatively, the reaction of glucose with glucokinase can be interpreted with CS as a relatively high affinity intrinsic binding step ( $K_{d}$ = 0.63 mM) that involves only a small fraction (10%) of glucokinase molecules that equilibrate at a rate $k_{12}+k_{21}$ = 8.0 s^−1^. Application of Eq. (15) yields $K_{d,\mathrm{app}}$ = 6.3 mM, which is identical to the value derived from IF, as expected, but that in this case greatly underestimates the intrinsic binding affinity of glucose for glucokinase. The two interpretations of glucose binding to glucokinase predict the same value of the apparent binding constant, which confirms that equilibrium measurements would be unable to assign a mechanism of binding. The two mechanisms differ significantly in predicting the intrinsic binding affinity of glucose for glucokinase, which is > 20-fold higher in the case of CS. The difference may have important implications for drug design. What value should be used to compare predictions of computational methods and binding constants measured experimentally at equilibrium, such as $K_{d,\mathrm{app}}$? The same interaction produces three distinct binding constants: CS predicts a value of 0.63 mM for the binding of glucose to the E form of glucokinase, representing 10% of the macromolecules in solution; a value of 6.3 mM, 10-fold higher than the intrinsic binding constant predicted by CS, measures the apparent equilibrium binding constant of glucose to glucokinase that would be measured by equilibrium titrations; finally, IF predicts an even larger value of 14 mM for the intrinsic binding constant of glucose to glucokinase before the complex rearranges into a more stable product. The value of 6.3 mM measured at equilibrium would be close to the intrinsic binding constant predicted by IF, but 10-fold higher than that predicted by CS. Therefore, a computational model of the glucose-glucokinase interaction based on the value of 6.3 mM measured at equilibrium would be fairly accurate if binding takes place according to IF, but quite inaccurate if binding takes place according to CS. This is valuable insight for a mechanism that remains controversial. Glucokinase was originally assumed to bind glucose at a single site according to IF,^54^ and crystal structures of the free and bound forms [Fig. 3(B)] support a sharp conformational transition “induced” by glucose binding.^55,56^ In contrast, kinetic measurements support CS^73^ and the existence of alternative conformations in equilibrium prior to the binding of any ligands.^74^ Recent X-ray structures of glucokinase also imply that there is little conformational change upon ligand binding to the enzyme, consistent with the predictions of CS in Eq. (12).^55^ Hence, the large conformational change induced upon glucose binding to the protein documented by structural biology [Fig. 3(B)] is inconclusive because it can be interpreted as the transition from E to E′X according to IF, or the transition from $E^{*}$ to EX according to CS. Similar inconclusive claims about the validity of IF have been made for the Mycobacterium tuberculosis methionyl-tRNA synthetase from comparison of large structural changes between free and bound forms of the enzyme.^75^ Multiple structures of a macromolecule in the free or bound forms are necessary to rigorously assign a binding mechanism in terms of IF or CS, as shown in the case of Na^+^ binding to thrombin [Fig. 5(B)]. The case for IF in glucokinase has resurfaced recently on the basis of the kinetic cooperativity of this enzyme and the need for an interpretation of the kinetic mechanism that involves both IF and CS in the reaction cycle.^76^ The argument is somewhat distinct from our discussion given that the basic equivalence in Eq. (33) may not apply to systems working far from equilibrium and under kinetic turnover. Furthermore, kinetic cooperativity requires a reaction scheme with at least two binding steps.^24,77–79^

**FIG. 8.**
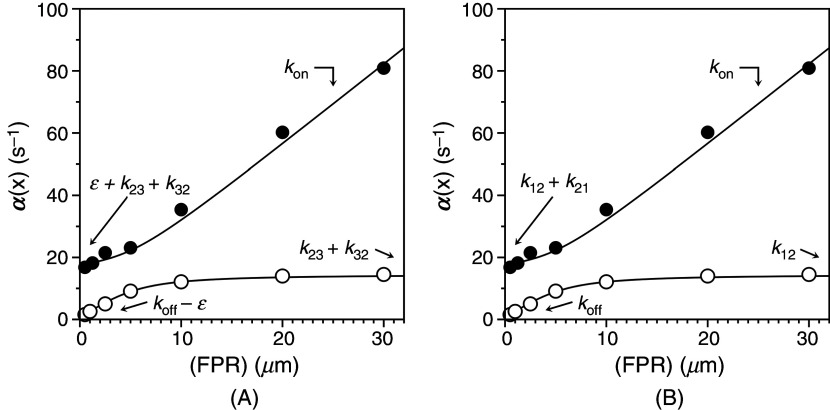
Rates of relaxation for FPR binding to thrombin^59^ under experimental conditions of: 50 mM Tris, 200 mM ChCl, 0.1% PEG8000, pH 8 at 10 °C. The fast relaxation increases linearly with FPR at high concentrations and reflect the binding interaction. The slow relaxation increases hyperbolically with FPR and can be interpreted with either IF or CS. (A) When the data are interpreted with IF, the best-fit parameter values are: $k_{\mathrm{on}}^{\text{IF}}$ = 2.6 ± 0.1 *μ*M^−1^s^−1^, $k_{\mathrm{off}}^{\text{IF}}$ = 3.6 ± 0.1 s^−1^, $k_{23}$ = 12 ± 1 s^−1^, $k_{32}$ = 2.6 ± 0.1 s^−1^. (B) The mathematically identical interpretation in terms of CS gives best-fit parameter values: $k_{\mathrm{on}}^{\mathrm{CS}}$ = 2.6 ± 0.1 μM^−1^s^−1^, $k_{\mathrm{off}}^{\mathrm{CS}}$ = 0.50 ± 0.02 s^−1^, $k_{12}$ = 14.6 ± 0.5 s^−1^, $k_{21}$ = 3.1 ± 0.3 s^−1^. Note how the interpretation of the lower limit of the fast relaxation and the two limits of the slow relaxation are widely different in the two mechanisms. Importantly, CS allows for a direct assessment of the individual rate constants in the kinetic scheme [Eq. (12)] by simple inspection of the plot. Adapted with permission from Ref. 59.

The basic equivalence in Eq. (33) also informs the interpretation of ligand binding to the active site of the clotting enzyme thrombin [Figs. 8(A) and 8(B)]. The two relaxations measured experimentally are shown in the same plot to illustrate how the limiting value of the fast relaxation at low ligand concentration is only slightly above the asymptotic value of the slow relaxation. The profile is consistent with both IF [Fig. 8(A)] or CS [Fig. 8(B)], and the two models give indistinguishable fits of experimental data.^59^ The two well-defined relaxations enable resolution of the four independent parameters in the kinetic scheme. Interpretation of the data in terms of IF [Fig. 8(A)] yields best-fit parameter values: $k_{on}$= 2.6 ± 0.1 *μ*M^−1^s^−1^, $k_{\mathrm{off}}$ = 3.6 ± 0.1 s^−1^, $k_{23}$= 12 ± 1 s^−1^, $k_{32}$ = 2.6 ± 0.1 s^−1^. The mathematically equivalent interpretation in terms of CS [Fig. 8(B)] gives best-fit parameter values: $k_{on}$= 2.6 ± 0.1 *μ*M^−1^s^−1^, $k_{\mathrm{off}}$ = 0.50 ± 0.02 s^−1^, $k_{12}$ = 14.6 ± 0.5 s^−1^, $k_{21}$= 3.1 ± 0.3 s^−1^. Again, application of Eq. (33) yields the equivalence

$$E\begin{array}{c} 2.6\;{\mu M^{-1}s}^{-1}x\;\\ \;⇄\;\\ 3.6\;s^{-1}\; \end{array}EX\begin{array}{c} 12\;s^{-1}\\ \;⇄\;\\ 2.6\;s^{-1} \end{array}E\prime X=E^{*}\begin{array}{c} 14.6\;s^{-1}\\ \;⇄\;\\ 3.1\;s^{-1} \end{array}E\begin{array}{c} 2.6\;{\mu M^{-1}s}^{-1}x\\ \;⇄\;\\ 0.50\;s^{-1} \end{array}EX.$$ (35)

When binding of FPR to thrombin is interpreted with IF, the intrinsic affinity is relatively high ( $K_{d}$ = 1.4 *μ*M) due to a near diffusion-controlled value of $k_{on}$ and is increased further, nearly fivefold, by a conformational rearrangement of the complex that equilibrates with a rate of $k_{23}+k_{32}$ = 14.6 s^−1^. The value of $K_{d,\mathrm{app}}$ = 0.25 *μ*M that would be measured from equilibrium titrations overestimates the affinity of the intrinsic binding interaction by nearly sixfold. The alternative but mathematically equivalent interpretation of the interaction CS results in a significant intrinsic binding affinity ( $K_{d}$ = 0.19 *μ*M) that involves over 80% of the thrombin molecules that preexist in equilibrium with a conformation that does not bind FPR, with the two conformations reaching equilibrium at a rate $k_{12}+k_{21}$ = 17.7 s^−1^. The predicted value of $K_{d,\mathrm{app}}$ = 0.25 *μ*M guarantees complete equivalence of the two mechanisms in the interpretation of equilibrium titrations and does not differ much from the value of the intrinsic binding constant because of the large fractions of thrombin in the E form. Unlike the case of glucokinase, IF and CS in thrombin envision a fairly rapid binding step that is preceded (CS) or followed (IF) by a rapid conformational transition. The main difference between the two mechanisms resides in the value of $k_{\mathrm{off}},$ which is nearly 10-fold faster in IF than CS, with a predicted value of the intrinsic binding affinity that is considerably weaker for IF. Again, the difference poses a challenge to computational biology.

An important special case of the equivalence in Eq. (33) is the reaction scheme that describes the irreversible binding interaction of a ligand with its macromolecule, i.e.,

$$E\begin{array}{c} k_{on}x\\ \;⇄\;\\ k_{\mathrm{off}} \end{array}EX\begin{array}{c} k_{23}\\ \to \end{array}E\prime X=E^{*}\begin{array}{c} k_{23}\\ \;⇄\;\\ k_{\mathrm{off}} \end{array}E\begin{array}{c} k_{on}x\\ \to \end{array}EX.$$ (36)

There are numerous examples in the pharmacology literature of such inhibitors,^51,80^ with aspirin and penicillin being among the most widely known.^81,82^ Serine protease inhibitors, or serpins,^83^ carry out their important biological function by irreversibly inactivating target proteases. The mechanism of inhibition is typically assumed to obey the IF mechanism [left reaction in Eq. (36)] where a reversible complex forms first and is then converted to a stable, irreversible final product.^84,85^ This widely accepted mechanism of inhibition is mathematically equivalent to an alternative CS mechanism where binding takes place irreversibly only to the E form of the macromolecule [right reaction in Eq. (36)]. As for the case in Eq. (33), IF and CS need to be critically examined before any conclusion can be drawn on the mechanism of recognition. This brings about the need for experimental strategies that distinguish between IF and CS.

## VIII. DISTINGUISHING BETWEEN IF AND CS

The demonstration that a slow relaxation increasing hyperbolically with the ligand concentration is consistent with both IF and CS calls into questions previous assignments based on the rapid equilibrium approximation^28^ and creates a need for strategies that can distinguish between IF and CS from analysis of experimental data. The need is made more acute by the complete mathematical equivalence of the two mechanisms according to Eq. (33).^59^ Unlike IF, CS can never be disproved *a priori* as a mechanism of ligand binding. Indeed, CS is always sufficient to explain the underlying kinetics and becomes necessary when the relaxation decreases hyperbolically with the ligand concentration. This is the case of cytochromes P450 that display remarkable plasticity in their ability to bind substrates and catalyze a broad array of chemical reactions.^86–89^ Strong evidence exists that such proteins bind ligands according to CS. In the case of cytochrome P450 P19A1, a detailed kinetic analysis using stopped flow has revealed the complexity of the recognition mechanism of three distinct ligands, i.e., androstenedione, testosterone, and 7-hydroxyflavone. A global fit of the entire kinetic traces supports CS and, in the case of testosterone, a multi-step binding mechanism such as the one in Eq. (42).^86^ The glucokinase regulatory protein plays an essential role in glucose homeostasis by acting as a competitive inhibitor of glucokinase and triggering its localization to the hepatocyte nucleus upon glucose deprivation. A small-molecule inhibitor of this protein-protein interaction utilizes CS by binding to a conformation of the regulatory protein that accounts for only 3% of the total population.^90^ CS is the driving force in the allosteric transition of Gal3p, an allosteric monomeric protein that activates the GAL genetic switch of *Saccharomyces cerevisiae* in response to galactose, which may have implications in other signaling pathways involving monomeric proteins.^91^ The chaperonin-containing t-complex polypeptide 1 assists protein folding in an ATP-dependent manner. Rapid kinetics experiments reveal a burst phase whose rate decreases with increasing ATP concentration, thereby proving that CS plays a key role in this system before hydrolysis.^92^ Additional systems that directly disprove IF with relaxation rates decreasing with the ligand concentration include alkaline phosphatase,^93^ glucokinase,^73^ the immunoglobulin IgE,^94^ and DNA in its B to Z transition.^95^ Several trypsin-like proteases feature kinetic signatures consistent with CS and structural evidence of alternative conformations in preexisting equilibrium in the free form.^22,60,64,71,96–99^ Similar observations have been reported recently for the flaviviral NS2B-NS3 protease.^100^ More generally, the immune system exploits CS as a strategy to diversify the repertoire of antigen specificities.^101^ Conformational rearrangements in antibody antigen recognition are essential events where kinetic discrimination of isomers expands the universe of combinations.

Structural information on the free and bound forms of the macromolecule is often of diagnostic value: evidence of multiple conformations in the free form [Fig. 4(B)] supports CS,^102^ and multiple conformations in the bound form support IF.^103^ When structural information is not available or inconclusive, the distinction between the two mechanisms must be made from the kinetic signatures of ligand binding. So far, our treatment of binding mechanisms has relied upon conditions where the ligand is in large excess over the macromolecule. These conditions are known as pseudo-first-order in the ligand concentration and are the most common encountered in practice because they are relatively easy to obtain experimentally and optimize the signal-to-noise ratio. It is instructive to analyze the behavior of the system under conditions where the ligand is no longer in excess over the macromolecule, a situation long used in enzyme kinetics for single turnover assays.^93,104^ We start with the simple lock-and-key mechanism and analyze the behavior of the system when the macromolecule is in excess compared to the ligand.^105^ In this case, the kinetic scheme is analogous to Eq. (9), i.e.,

$$X_{\;}\begin{array}{c} k_{on}e\\ \;⇄\;\\ k_{\mathrm{off}} \end{array}EX,$$ (37)

where e denotes the concentration of free macromolecule that does not change significantly when the ligand X binds. The system is under pseudo-first-order conditions in the macromolecule concentration. The rate constants $k_{on}$ and $k_{\mathrm{off}}$ are the same as in Eq. (9) and can be determined by measurements of the relaxation of the system to equilibrium as a function of the perturbing variable e, i.e.,

$$\alpha (e)=k_{on}e+k_{\mathrm{off}}.$$ (38)

The relationship above is analogous to Eq. (10) and introduces a linear relation between the rate of relaxation to equilibrium and the concentration e. Consequently, measurements carried out with excess ligand or macromolecule yield the same linear dependence of the relaxation. When the same conditions of excess macromolecule apply to IF, the relevant reaction scheme becomes

$$X\begin{array}{c} k_{on}e\\ \;⇄\;\\ k_{\mathrm{off}} \end{array}EX\begin{array}{c} k_{23}\\ \;⇄\;\\ k_{32} \end{array}E\prime X$$ (39)

and is similar to Eq. (11). Accordingly, the two relaxations assume the same form as Eqs. (19a) and (19b) with the independent variable x replaced by e. As for the lock-and-key mechanism, measurements of the relaxations for IF will not change when excess ligand is replaced by excess macromolecule. However, this is not the case for CS. When the ligand is in excess, the macromolecule transitions from the free to the fully bound state as the ligand concentration increases and its distribution in the preexisting equilibrium is drastically affected. When the macromolecule is in excess, the total amount bound to the ligand hardly affects the initial distribution between alternative conformations, and the preexisting equilibrium is no longer detected experimentally. Under conditions where the macromolecule is in excess, the preexisting equilibrium in Eq. (12) is not significantly perturbed upon ligand binding and therefore drops from the kinetic scheme that becomes identical to Eq. (37) for a simple lock-and-key mechanism. When binding obeys CS with excess ligand, it will obey lock-and-key with excess macromolecule. Therefore, IF and CS cannot be distinguished under conditions of excess ligand but become easily distinguishable under conditions of excess macromolecule. This feature of the two mechanisms can be used as a diagnostic test when measurements under excess ligand show a hyperbolic increase in the slow relaxation as a function of x. A comparison of rapid kinetics with excess macromolecule vs excess ligand is expected to produce no differences for IF, but should turn the hyperbolic increase with excess ligand into a straight line or constant value with excess macromolecule for CS.^106^ This test has been used to validate IF or CS in various systems,^63,93,105,107–109^ starting with the pioneering work of Halford with E. coli alkaline phosphatase.^93,104^ Galletto *et al.*^63^ used this approach to distinguish between IF and CS in the analysis of metA binding with DNAC after their simulations revealed that CS could produce a relaxation increasing hyperbolically with the ligand concentration as seen for IF. Measurements with excess thrombin over FPR prove that the relaxations in Fig. 8(A) are due to CS and not IF.^107^ Measurements with excess thrombin over the irreversible inhibitor H-D-Phe-Pro-Arg-CH_2_Cl (PPACK) also prove that binding takes place according to CS [right portion of Eq. (36)] rather than IF [left portion of Eq. (36)].^109^ On the other hand, measurements with excess antithrombin over heparin prove that heparin binding to antithrombin obeys IF and not CS.^108^ No diagnostic measurements have been carried out for glucokinase [Fig. 3(A)] by comparing pseudo-first-order conditions of ligand and macromolecule. If feasible, such measurements would help solve some of the existing controversies about the mechanism of binding. In general, a great deal of existing experimental data interpreted according to IF from measurements carried out with excess ligand would benefit from validation with measurements with excess macromolecule.

The mathematical analysis discussed in Eqs. (37)–(39) has been developed by Gianni *et al.*^105^ A more detailed mathematical treatment has been presented recently by Paul and Weikl^106^ who analyzed the behavior of a system where ligand and macromolecule are present in variable ratios. They found that the kinetic equations of IF, or of the lock-and-key mechanism, depend on the sum of the total concentrations of ligand and macromolecule^93^ and are therefore invariant under conditions that alter the relative proportion of these components but not their total balance. That is not the case for CS, where the preexisting equilibrium of the macromolecule is perturbed only when the ligand is in excess.^93^ The analysis of Paul and Weikl generalizes the kinetic equations for the case of all ligand and macromolecule concentrations. For IF, they find that the slow relaxation is symmetric around a minimum in the ligand concentration $x_{\mathrm{tot}}=e_{\mathrm{tot}}-K_{d,\mathrm{app}}$, where $x_{\mathrm{tot}}$ and $e_{\mathrm{tot}}$ refer to the total concentrations of ligand and macromolecule. Obviously, the minimum is only observed for $e_{\mathrm{tot}}>K_{d,\mathrm{app}}$, which puts limitations on practical applications. A defining feature of the dependence of the slow relaxation on $x_{\mathrm{tot}}$ rather than x is that the entire function is symmetric around this minimum. In the case of CS, the minimum also depends on the relative values of $k_{12}$ and $k_{\mathrm{off}}$ and the function is not symmetric around the minimum. At high enough values of $e_{\mathrm{tot}}$, the relaxation is significantly faster at low $x_{\mathrm{tot}}$ values, thereby allowing for a simple distinction with the symmetric curve predicted by IF. The experimental case used to demonstrate the complex behavior of the slow relaxation in the general case is that of recoverin binding to rhodopsin studied by NMR, calorimetry, and stopped flow.^110^ The authors conclude that protein dynamics in free recoverin limit the overall rate of binding for this interaction and is entirely consistent with CS.

Although the strategy of comparing measurements with excess ligand and excess macromolecule is informative,^63,105,106^ it may find limited application in practice. Large concentrations of macromolecule may be limited by availability and often produce complications like aggregation or loss of stability. Increasing the ratio of macromolecule over ligand also weakens the amplitude of spectroscopic signal and adds significant error to the kinetic traces. Applicability of the approach based on the general equations involving the total concentrations of ligand and macromolecule offers a clear distinction between IF and CS only for very large concentrations of the macromolecule such that $e_{\mathrm{tot}}>K_{d,\mathrm{app}}$. For example, in the interaction of two disordered protein domains ACTR and NCBD, two phases can be distinguished when the experiment is performed under pseudo-first-order conditions for ACTR, but the same observation cannot be made easily under pseudo-first-order conditions for NCBD due to the high total fluorescence with excess NCBD.^105^ The validity of IF was surmised in this case by assuming that the resulting single phase observed under excess NCBD was the average of the two phases under excess ACTR, vouching for an IF mechanism, as originally assumed.^111^ The conclusion is not rigorous, and the example documents the difficulty of studying a system interchangeably with excess ligand or macromolecule.

Alternative approaches to resolve the ambiguity between IF and CS have been proposed in terms of global fit analysis^112^ or native and ion mobility mass spectrometry.^113^ Other approaches have argued that IF and CS can be distinguished from the effect of mutations far from the binding site for the ligand, which affect mainly $k_{\mathrm{off}}$ for IF and $k_{on}$ for CS,^114^ but the conclusion is not general. Yet other approaches have ruled out *a priori* CS in the case of enzymes with lid-gated active sites such as ribokinase,^115^ adenosine kinase,^116^ and glucokinase,^103^ where closure of the lid must follow the binding step to trigger catalysis.^117^ This argument is easily refuted because the closed conformation of the enzyme may preexist in equilibrium with the open one, as pointed out by Wolfenden.^118^ That would generate a three-step mechanism encompassing both CS and IF (see Sec. X), similar to that envisioned by Galletto *et al.* for MANT-ADP binding to the DnaC protein.^63^ More convincing support for IF comes from enzymes that undergo a large conformational change to trap substrate in a tight protein cage to promote catalysis.^119,120^ This is the case of triosephosphate isomerase,^121^ glycerol phosphate dehydrogenase^122^ and orotidine 5'-monophosphate decarboxylase.^123^ However, the kinetic mechanism of catalysis also requires an inactive open form of the enzyme to coexist in equilibrium with the active closed form prior to ligand binding, as envisioned by CS.^119^ Selective binding of the ligand to the active form is then the trigger for expression of the full transition-state binding energy. In general, mechanisms of enzyme catalyzed reactions should consider multiple conformations in the free form when defining the steps leading to substrate binding in the transition state instead of limiting the description to IF transitions from poorly active to fully catalytic conformations.^119^ Finally, IF and CS can be detected as selected pathways or fluxes in extended kinetic mechanisms that encompass both mechanisms as special cases,^76,124–126^ but it is unclear if such approaches have general applicability or are even necessary for the interpretation of systems where only a single relaxation is accessible experimentally under conditions of excess ligand, which is the most common situation encountered in practice. Other, more effective strategies to distinguish between CS and IF make use of the mathematical underpinnings described in Secs. V and VI. A close inspection of Fig. 8 shows that the value of $\alpha_{2}(0)$ is a complicated function of the rate constants in the case of IF but defines $k_{\mathrm{off}}$ in the case of CS. If such a parameter can be determined experimentally from independent measurements, then a direct test of the validity of CS can be obtained. An elegant application of this strategy has been presented recently for sugar binding to LacY^127^ and has helped assign CS as the mechanism of recognition. The authors were able to measure $k_{\mathrm{off}}$ from competition experiments and prove that the value coincided with the value of $\alpha_{2}(0)$ in the plot of the slow relaxation measured experimentally. In general, the value of $\alpha_{2}^{\;}\left(\infty \right)$ measures a property of the free macromolecule for CS or of the bound complex for IF and is expected to change with different ligands for IF but not for CS. Therefore, a set of carefully designed measurements with different ligands under identical solution conditions may easily discriminate between IF and CS, even under conditions of excess ligand and with a single slow relaxation available experimentally. If the system obeys CS, then the slow relaxation must saturate at the same value for different ligands. This is because the value of $\alpha_{2}\left(\infty \right)=k_{12}$ measures the rate for the $E^{*}\to E$ transition, which is a property of the free macromolecule, independent of the particular ligand used. A value of $\alpha_{2}\left(\infty \right)$ that changes with different ligands most likely measures the sum $k_{23}+k_{32}$ of the IF mechanism, which will obviously depend on the nature of bound complex formed and rearranged. The data shown in Fig. 5 demonstrate that CS is definitely at play for FPK, but FPR likely obeys the same mechanism because its slow relaxation saturates at the same value as the lower limit of FPK.^59^ Similar conclusions have been drawn for the P-type ATPases when using metal-fluoride complexes^62^ and should have been considered for recent host-guest studies reporting a switch between IF and CS by changing the ligand.^128^

A new and promising way to assign mechanisms of ligand binding has emerged from application of NMR. Molecular recognition according to IF or CS involves preferred binding of a molecule to one of several conformations in solution in the context of the emerging view of the macromolecule as a conformational ensemble^43,129,130^ promoted by NMR,^131^ X-ray crystallography,^102^ single molecule spectroscopy,^132^ and cryo-EM.^133,134^ The bound structure can be selected from the ensemble of interconverting conformations in CS or may be generated anew in IF. There is growing appreciation that the link between protein dynamics and ligand concentration can shift a binding mechanism between IF and CS^43,125,135–137^ and that both mechanisms can be detected and distinguished under proper conditions, i.e., using solution NMR spectroscopy that exploits a methyl-transverse relaxation-optimized spectroscopy effect and selective isotope-labeling methodologies.^138^ Such advanced NMR experiments indicate that conformational changes can occur in the free form of the macromolecule (CS) or in the bound complex (IF), but CS requires transition times for ligand binding and unbinding that are small compared to the dwell times of proteins in different conformations, and the reverse is true for IF. This separation of time scales and ordering of events can be determined from relaxation rates and effective binding and dissociations events measured in advanced NMR^139^ or even single molecule Förster resonance energy transfer experiments.^132^ Systems that have been assigned to IF include the Mycobacterium tuberculosis β-lactamase that is mostly rigid in the free form, as established by ^15^N relaxation experiments, but becomes more dynamic upon binding of the antibiotic avibactam.^140^ IF has been invoked as a mechanism to optimize molecular switches such as aptamers that change their conformation upon target binding to benefit applications in biotechnology and synthetic biology,^141^ as well as a mechanism for the Asp symporter opening upon Na^+^ binding,^142^ for an archaeal homolog of the excitatory amino acid transporter involved in glutamatergic synaptic transmission in the mammalian central nervous system^143^ and for selective inhibitors of the FK506-binding protein 51.^144^ A number of systems, on the other hand, are more consistent with CS. Both bacterial and human Hsp70 chaperones interact with client proteins by selecting the unfolded state from a preexisting array of interconverting structures, suggesting a conserved mode of client recognition among Hsp70s and highlighting the importance of CS in this recognition.^138,145^ Ligand binding to adenylate kinase^146^ and activation of the A2A adenosine G-protein-coupled receptor obey CS.^147^ In general, molten globule active sites take advantage of CS.^148^ The cytokine IL-2 undergoes an open-closed pre-equilibrium revealed by NMR that controls binding to its receptor IL2-Rα and offers opportunities to be manipulated for drug discovery.^149^ A similar open-closed pre-equilibrium is observed in the α/β-hydrolase MenH^150^ and human glutathione transferase, a major detoxification enzyme and key regulator of cell proliferation.^151^ Open, closed, and “nucleotide-binding” states preexists for Klentaq1.^152^ E7 from human papillomavirus, a prototypic viral oncoprotein and a model intrinsically disordered protein, has an immunodominant epitope within a “hinge” region between the N-terminal intrinsically disordered and the C-terminal globular domains that has at least two populations separated by a high-energy barrier as determined by NMR. Presentation of this viral epitope by the antigen-presenting cells takes place in the low populated conformation.^153^ CS has been documented in the host-guest encapsulation behavior of a new enzyme-mimetic metal-ligand host by NMR studies and kinetic traces where increasing the concentration of the guest inhibits the rate of host-guest relaxation.^154^ Similar observations unequivocally supporting CS have been reported recently for macrocyclic receptors by NMR and rapid kinetics studies.^155^

## IX. THE LINKAGE SCHEME

Both IF and CS extend the original lock-and-key model by incorporating conformational transitions that either precede or follow the binding step. The extension is noteworthy and captures the plasticity of biological macromolecules that is reflected in a relaxation that saturates with increasing ligand concentration. However, both IF and CS have intrinsic limitations because the former does not allow for conformational heterogeneity in the free form of the macromolecule and the latter precludes this plasticity to occur in the bound form. From a statistical thermodynamic standpoint, the bound conformation $E^{\prime }X$ in IF should also exist in the free form as $E^{\prime }$, even if in minuscule amounts. By the same argument, the free conformation $E^{*}$ in CS should be able to bind the ligand as $E^{*}X$, even if with minuscule affinity. A polarized division of ligand binding mechanisms in terms of the simple IF and CS fails to capture the complexity of numerous biological macromolecules. The case for a close interplay between IF and CS is supported by numerous arguments and systems. A particularly important example given its general relevance in biology is provided by the rate acceleration of enzyme catalyzed reactions due to stabilizing interactions between the protein and the transition state, which is bound with higher specificity than substrate. An important contribution to this effect comes from the conversion of multiple forms of the enzyme in the free form to fewer, bound rigid forms. Hence, catalysis is optimized by conversion of the initial complex into a more catalytically competent transition state, as envisioned by IF, and collapse of the entropically rich ensemble of conformations in the free form upon binding of substrate, as envisioned by CS.^119^ The case for a more general scheme that incorporates both IF and CS becomes of the essence. This leads to consideration of the following linkage scheme:

E*kon*x ⇄ koff*E*Xk12⇃↾k21 k43⇃↾k34Ekonx ⇄ koffEX, (40)

where the macromolecule is assumed to exist in two alternative conformations, each capable of binding the ligand at a single site.^15,104^ The contribution of IF is evident from the $E^{*}\;⇄\;\;E^{*}X\;⇄\;\;EX$ pathway, whereas the contribution of CS is shown by the alternative $E^{*}\;⇄\;\;E\;⇄\;\;EX$ pathway. The scheme has been the subject of intense investigation. The equilibrium properties of the scheme are well known and form the basis of linkage thermodynamics.^25,30^ Botts and Morales provided an exact analytical solution for its properties at steady state,^78^ Eigen recognized it as the simplest mechanism encompassing both IF and CS as special cases,^15^ Wyman analyzed its linkage properties in terms of the “turning wheel” model,^156^ Frieden discussed the “hysteresis” properties,^79^ and Hill provided a comprehensive study of the scheme at steady state and far from equilibrium in terms of fluxes and specific paths involving the four species.^24^ Recent discussions of the linkage scheme have been presented in terms of mutational analysis,^114^ fluxes,^136^ and diffusion-controlled reactions.^157^ Numerous systems have provided compelling evidence of the importance of this scheme. Agitoxin-2 from scorpion venom is a potent blocker of K^+^ channels. Analytical imaging of binding to the KcsA channel in real time using high-speed atomic force microscopy is consistent with the properties of a linkage scheme where IF predominates.^158^ A mechanism involving both IF and CS as in the linkage scheme has been invoked for the binding of MutL to a UvrD monomer–DNA complex,^159^ the U1A-RNA interaction,^160^ and the binding of an anticancer drug to c-Src kinase.^161^ Riboswitches bind ligand according to IF and CS depending on solution conditions.^162,163^ Recent single-molecule experiments support a dominant role for IF and the linkage scheme^164^ but ligand-detected Carr-Purcell-Meiboom-Gill relaxation dispersion experiments make a strong case for CS and as a valuable diagnostic tool for the characterization of binding mechanisms by NMR.^165^ Fluorescence spectroscopy in a microfluidics channel shows that molecular recognition of α-chymotrypsin at two different pH values follows two distinctly different pathways in the linkage scheme, i.e., IF or CS, at high or low ligand concentrations, respectively.^166^ Binding-induced folding under unfolding conditions switches progressively from CS to IF.^167^ The need for more complex mechanisms that go beyond the simple IF and CS has also emerged from crystallographic and NMR studies on the mitochondrial Tom20 protein–presequence interaction and possibly for other promiscuous recognitions of signal peptides by the RP54/Ffh and SecA proteins.^168^ Maltose-binding protein shows structural evidence of conformational transitions preceding and following the binding step.^131,169^

The linkage scheme in Eq. (40) may foster the conclusion that IF and CS split the functional complexity of the entire scheme. This conclusion has cast IF and CS as special cases that dominate the functional behavior of the linkage scheme based on the ligand concentration, with IF prevailing at high ligand concentrations and CS prevailing at low ligand concentrations.^76,125,126,135,136,157,166^ Future studies should reconsider these widely accepted conclusions on the different contributions of IF and CS to the linkage scheme in view of their mathematical equivalence [Eq. (33)]. In fact, it is unlikely that IF and CS split their contribution to the kinetic properties of the linkage scheme given that CS includes IF as a special case^59^ and alone recapitulates most of the functional repertoire of the entire scheme.^72^ There is no functional symmetry or equal partitioning between IF and CS within the linkage scheme, which provides additional evidence of the dominance of CS as a mechanism of ligand binding.

There are four species and eight parameters in the linkage scheme, seven of which are independent due to detailed balancing.^24,78^ Conservation of mass limits the number of independent species to three and gives an equal number of independent relaxations. Two such relaxations refer to the binding interactions $E^{*}+X\;⇄\;\;E^{*}X$ and E+X ⇄  EX, and eventually increase linearly with x. Only one relaxation is saturable and reflects the interplay between binding and conformational transitions. Hence, the linkage scheme produces a single saturable relaxation like the simpler IF and CS schemes. This relaxation may also happen to be the only one accessible to experimental measurements if the two binding interactions are too fast. In general, when only a single saturable relaxation is available, the linkage scheme should be considered as a plausible interpretation of experimental data, especially if the predictions of the simpler IF and CS are not confirmed experimentally. The analytical expressions for the three relaxations of the linkage scheme require solution of a cubic expression of the eigenvalues of the underlying 4 × 4 matrix associated with the scheme, but they are algebraically cumbersome and of little practical use. However, the limiting values of the saturable relaxation define the salient properties of the linkage scheme. These values are^72^

$$\alpha_{1,2}(0)=\frac{1}{2}\{k_{\mathrm{off}}+k_{\mathrm{off}}^{*}+k_{34}+k_{43}\pm \sqrt{{\left(k_{\mathrm{off}}+k_{\mathrm{off}}^{*}+k_{34}+k_{43}\right)}^{2}-4\left(k_{\mathrm{off}}k_{\mathrm{off}}^{*}+k_{\mathrm{off}}^{*}k_{34}+k_{\mathrm{off}}k_{43}\right)}\}$$ (41a)

$$\alpha_{1,2}\left(\infty \right)\approx k_{on}x\;or\;k_{on}^{*}x$$ (41b)

$$\alpha_{3}(0)=k_{12}+k_{21}$$ (41c)

$$\alpha_{3}(\infty )=k_{34}+k_{43}.$$ (41d)

It is understood that the values of $\alpha_{1,2,3}(0)$ must be ranked such that $\alpha_{1}\left(0\right)>\alpha_{2}\left(0\right)>\alpha_{3}\left(0\right),$ and only the value of $\alpha_{3}(0)$ should be compared with the limit $\alpha_{3}(\infty )=k_{34}+k_{43}$ to determine if the saturable relaxation increases, decreases, or remains constant as a function of the ligand concentration. The limits for IF and CS are obtained as special cases from Eqs. (41a)–(41d) by removing all rate constants pertaining to CS or IF, respectively. The decoupling of the two limiting values of the saturable relaxation allow for greater flexibility in the analysis of experimental data that may prove beneficial over the stricter predictions of the simpler CS scheme. For example, the linkage scheme does not require FPR and FPK in Fig. 5 to saturate at the same value of the relaxation for x→∞ because $\alpha_{3}(\infty )=k_{34}+k_{43}$ depends on the exchange $E^{*}X\;⇄\;\;EX$ that is a property of the complex, rather than on the $E^{*}\to E$ transition that is a property of the free macromolecule. However, in the absence of evidence of two linear relaxations, it is difficult to make a case for the greater mathematical complexity of the linkage scheme and the inability to resolve any of its rate constants [Eq. (40)] from analysis of experimental data. Interestingly, Galburt has recently shown that the saturable relaxation of the linkage scheme may show a minimum under certain conditions, a behavior that is not mathematically possible with either IF or CS. The minimum correlates with a transition in the flux between CS and IF pathways within the linkage scheme. If observed experimentally, such minimum would make a strong case in support of the linkage scheme, even in the absence of the two relaxations referring to the binding steps.^124^

## X. OTHER MECHANISMS OF BINDING

**FIG. 9.**
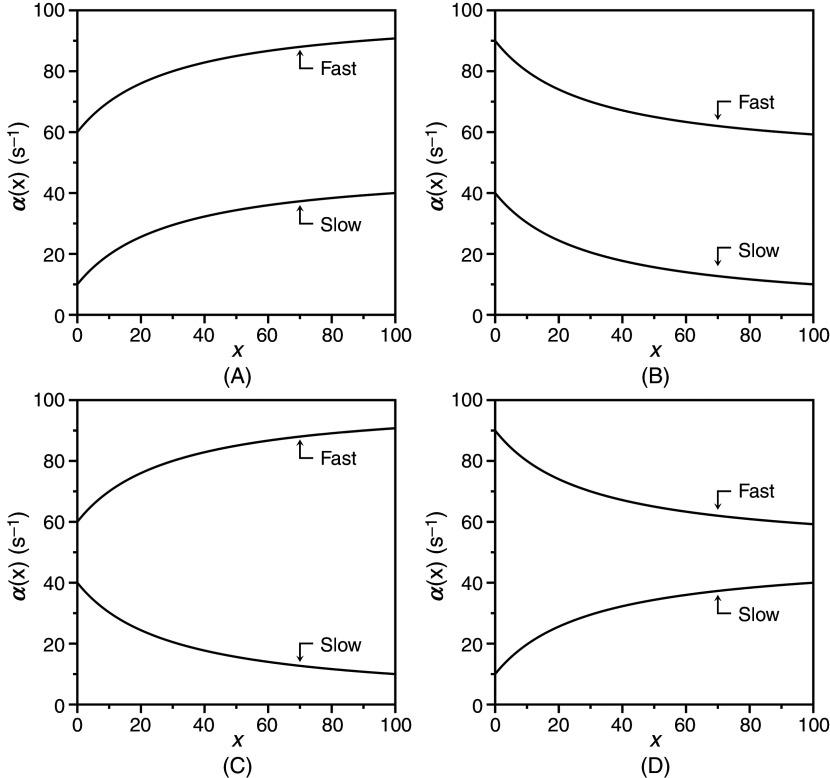
Four possible outcomes for a system obeying two saturable relaxations, fast and slow, that both (A) increase and (B) decrease, with (C) the fast relaxation increasing and the slow one decreasing or (D) vice versa. These scenarios map to the four quadrant plot in Fig. 10, where the log of the ratio α(∞)/α(0) for the slow relaxation is plotted vs that of the fast relaxation.

**FIG. 10.**
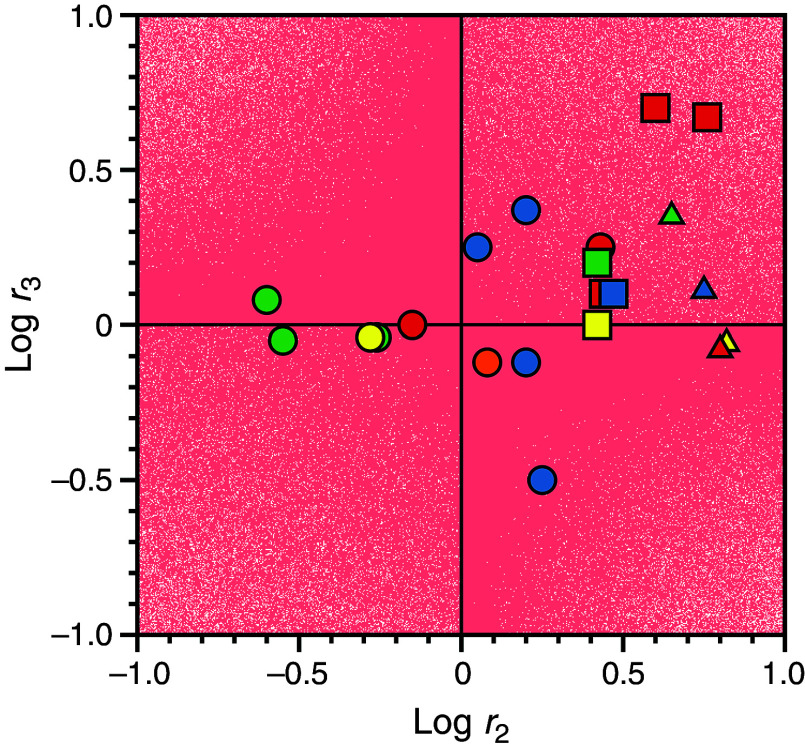
Four-quadrant plot of the log of the ratio α(∞)/α(0) for the slow relaxation, $r_{3}$, vs that of the fast relaxation, $r_{2}$ (see also Table I). Experimental data points refer to 13 different systems: IgE^94^ (green circles), IgG^170^ (green square), 3-hydroxybenzoate 6-hydroxylase^175^ (green triangle), DnaC^63^ (yellow circle), polymerase X^174^ (yellow square), CheA^172^ (yellow triangle), protein kinase A^171^ (red circles), K^+^-mediated G-quadruplex folding^178^ (red squares), 3-chloroacrylic acid dehalogenase^176^ (red triangle), DnaB^179^ (blue circles), histone deacetylase-like amidohydrolase^173^ (blue square), proline utilization A protein^177^ (blue triangle), ACTR, and CREB-binding protein^111^ (orange circle). The top left quadrant refers to the case where the fast relaxation decreases and the slow one increases [see also Fig. 9(D)] and contains a single data point from DNP-Ser binding to IgE SPE7.^94^ The top right quadrant refers to the case where both relaxations increase [see also Fig. 9(A)] and contains 55% of the experimental points. The bottom right quadrant refers to the case where the fast relaxation increases and the slow one decreases [see also Fig. 9(C)] and contains 23% of the cases. The bottom left quadrant refers to the case of both relaxations decreasing [see also Fig. 9(B)] and contains 18% of the cases. Red dots depict values generated by the kinetic scheme in Eq. (43) from a total of 5 million simulations of the relaxations in Eqs. (46a) and (46b). Rate constants were generated over a range of 6 orders of magnitude using the expression $k={1000e}^{-\omega }$, where ω is a normally distributed random number with μ = 0 and σ = 3. The kinetic scheme in Eq. (43) accounts for all possible outcomes in the plot. Adapted with permission from Ref. 72.

When a single, monotonic and saturable relaxation is measured experimentally, CS offers the simplest interpretation of the mechanism of binding. The improved time resolution of stopped flow instruments has made it possible to detect faster events and expand the number of relaxations accessible experimentally. In addition to better resolution of rapid binding components of the kinetic mechanism, recent systems have been able to resolve multiple saturable relaxations and detect kinetic profiles that cannot be accounted for by IF, CS, or even the linkage scheme. When two saturable relaxations are detected, four distinct scenarios must be considered: both relaxations increase with the ligand concentration [Fig. 9(A)], both decrease [Fig. 9(B)], the fast relaxation increases and the slow one decreases [Fig. 9(C)], or vice versa [Fig. 9(D)]. In all cases, the fast saturable relaxation exceed all values of the slow one. Figure 10 summarizes these different possibilities in the context of available experimental data. To facilitate comparison, the plot displays the log of the ratio α(∞)/α(0) for the slow saturable relaxation $\alpha_{3}(x)$ vs that of the fast saturable relaxation $\alpha_{2}(x)$. We hypothesize that the fastest relaxation $\alpha_{1}(x)$ is linked to the binding event and is too fast to measure. Experimental data refer to 13 different systems, i.e., immunoglobulins IgE^94^ and IgG,^170^ protein kinase A,^171^ DnaC,^63^ CheA,^172^ histone deacetylase-like amidohydrolase,^173^ polymerase X,^174^ 3-hydroxybenzoate 6-hydroxylase,^175^ 3-chloroacrylic acid dehalogenase,^176^ proline utilization A protein,^177^ ACTR and CREB-binding protein,^111^ G-quadruplex folding,^178^ and DnaB.^179^ With the exception of DNP-Ser binding to IgE SPE7,^94^ the experimental points populate all quadrants except the one in the upper left where the fast saturable relaxation decreases and the slow one increases with the ligand concentration. Both relaxations increase in 55% of the cases, both decrease in 18% of the cases, and the fast relaxation increases and the slow one decreases in 23% of the cases. A single instance is recorded where the fast relaxation decreases while the slow one increases. What mechanism explains the behavior of such systems and the distribution seen in Fig. 10? A suitable kinetic mechanism should feature at least two conformational transitions associated with two independent relaxations. Expansion of the number of free forms in pre-equilibrium from two to three, while allowing binding to only one of them, generates an extended CS scheme where two saturable relaxations can increase or decrease with x but never in a combination where the fast saturable relaxation decreases and the slow one increases. This extended model does not account for the profile shown in Fig. 9(D) or the data at the top left quadrant of Fig. 10. However, it is notable that this extended CS scheme is capable of reproducing the distribution observed experimentally in the majority of cases^72^ and explains why a decrease in the fast saturable relaxation rarely takes place in conjunction with an increase in the slow one (Fig. 10). On the other hand, extension of IF to include additional conformational transitions following the binding step generates saturable relaxations that always increase with the ligand concentration. Hence, IF cannot generate saturable relaxations that decrease with x, regardless of the number of conformational transitions that follow the binding step. As a result, an expanded IF mechanism only accounts for the case mapping to the upper right quadrant of the plot in Fig. 10 and captures half of the cases observed experimentally. The difference in kinetic profiles observed between expanding CS and IF supports the conclusion that functional complexity is achieved by increasing the conformational heterogeneity of the free form of the macromolecule, i.e., by increasing the number of preexisting conformations of the macromolecule from which the ligand selects the best fit. The contribution to kinetic complexity from increased heterogeneity of the bound forms is significantly smaller.

A simple combination of IF and CS produces a kinetic mechanism of considerable versatility that accounts for all cases reported in Fig. 10.^72^ Elimination of one binding step in the linkage scheme [Eq. (40)] has the interesting effect of removing one of the two relaxations reflecting binding but increasing the relaxations linked to conformational transitions. This is because linearization of the linkage scheme keeps the number of independent species and relaxations unchanged. The mechanism entails a preexisting equilibrium between two forms of the macromolecule, allowing selective binding to one of the forms and subsequent isomerization to a second bound form, i.e.,

$$E^{*}\begin{array}{c} k_{12}\\ \;⇄\;\;\\ k_{21} \end{array}E\begin{array}{c} k_{on}x\\ \;⇄\;\;\\ k_{\mathrm{off}} \end{array}EX\begin{array}{c} k_{34}\\ \;⇄\;\\ k_{43} \end{array}E^{\prime }X.$$ (42)

There are six independent parameters in the scheme and three independent species, leading to three non-zero eigenvalues in the 4 × 4 matrix associated with it. The general solution of the scheme is again a cubic expression as for the linkage scheme and algebraically cumbersome. However, the functional versatility of the scheme can be appreciated by a simplified version under the rapid equilibrium approximation where the binding step is assumed to be considerably faster than the conformational transitions $E^{*}\;⇄\;\;E$ and $EX\;⇄\;\;E^{\prime }X$. Under this assumption, the scheme contracts to

$$E^{*}\begin{array}{c} k_{12}\\ \;⇄\;\;\\ k_{21} \end{array}\langle E|EX\rangle \begin{array}{c} k_{34}\\ \;⇄\;\\ k_{43} \end{array}E^{\prime }X,$$ (43)

which is equivalent to the following two-step mechanism directly expanding Eq. (1), i.e.,

$$E_{1}\begin{array}{c} k_{12}\\ \;⇄\;\\ k_{21} \end{array}E_{23}\begin{array}{c} k_{34}\\ \;⇄\;\\ k_{43} \end{array}E_{4}.$$ (44)

The numbering in Eq. (44) has been modified to match the rate constants in Eq. (43) and to indicate that the species $E_{23}$ is the equilibrium distribution of the two individual species $E_{2}$ and $E_{3}$. The two independent relaxations associated with Eq. (44) are

$$\alpha_{2,3}=\frac{1}{2}\left\{k_{12}+k_{21}+k_{34}+k_{43}\pm \sqrt{{\left[k_{12}+k_{21}-k_{34}-k_{43}\right]}^{2}+4{k_{21}k}_{34}}\right\}.$$ (45)

Again, we note that the sums $k_{12}+k_{21}$ and $k_{34}+k_{43}$, measuring the rates at which the transitions $E_{1}\;⇄\;\;E_{23}$ and $E_{23}\;⇄\;\;E_{4}$ reach equilibrium, make a symmetric contribution to Eq. (45) and can be swapped without consequences. On the other hand, the term ${k_{21}k}_{34}$ makes a unique contribution to Eq. (45) that depends on rates that selectively deplete the $E_{23}$ intermediate. The fast relaxation in the extended scheme [Eq. (42)] is the same as the lock-and-key expression in Eq. (9). The two slower relaxations $\alpha_{2}\left(x\right)$ and $\alpha_{3}\left(x\right)$ associated with the conformational transitions are derived from Eq. (45) as

$$\alpha_{2}\left(f\right)=\frac{1}{2}\{k_{12}+k_{21}\left(1-f\right)+k_{34}f+k_{43}+\sqrt{{\left[k_{12}+k_{21}\left(1-f\right)-k_{34}f-k_{43}\right]}^{2}+4{k_{21}k}_{34}f(1-f)}\}$$ (46a)

$$\alpha_{3}\left(f\right)=\frac{1}{2}\{k_{12}+k_{21}\left(1-f\right)+k_{34}f+k_{43}-\sqrt{{\left[k_{12}+k_{21}\left(1-f\right)-k_{34}f-k_{43}\right]}^{2}+4{k_{21}k}_{34}f(1-f)}\}$$ (46b)

$$f=\frac{x}{K_{d}+x},$$ (46c)

where f measures the fractional satuaration of E within the rapid equilibrium exchange $\left\langle \langle E|EX\rangle \right\rangle$. The limiting values of these relaxations are

$$\alpha_{2}\left(0\right)=\mathrm{larger}\;of\;k_{12}+k_{21}\;or\;k_{43}$$ (47a)

$$\alpha_{3}\left(0\right)=\mathrm{smaller}\;of\;k_{12}+k_{21}\;or\;k_{43}$$ (47b)

$$\alpha_{2}\left(\infty \right)=\mathrm{larger}\;of\;k_{12}\;or\;k_{34}+k_{43}$$ (47c)

$$\alpha_{3}\left(\infty \right)=\mathrm{smaller}\;of\;k_{12}\;or\;k_{34}+k_{43}.$$ (47d)

Four cases must be considered depending on the values of the $\alpha_{2}\left(\infty \right)/\alpha_{2}\left(0\right)$ and $\alpha_{3}\left(\infty \right)/\alpha_{3}\left(0\right)$, i.e.,

$$\text{Case}1:r_{2}=\frac{k_{12}}{k_{12}+k_{21}}\;\mathrm{and}\;r_{3}=\frac{k_{34}+k_{43}}{k_{43}}\mathrm{fast}\;\mathrm{decreases},\;\mathrm{slow}\;\mathrm{increases}$$ (48a)

$$\text{Case}\;2:r_{2}=\frac{k_{12}}{k_{43}}\;\mathrm{and}\;r_{3}=\frac{k_{34}+k_{43}}{k_{12}+k_{21}}\mathrm{fast}\;\mathrm{increases},\;\mathrm{slow}\;\mathrm{decreases}$$ (48b)

$$\text{Case}\;3:r_{2}=\frac{k_{34}+k_{43}}{k_{12}+k_{21}}\;\mathrm{and}\;r_{3}=\frac{k_{12}}{k_{43}}\mathrm{fast}\;\mathrm{decreases}/\mathrm{increases},\;\mathrm{slow}\;\mathrm{decreases}/\mathrm{increases}$$ (48c)

$$\text{Case}\;4:r_{2}=\frac{k_{34}+k_{43}}{k_{43}}\;\mathrm{and}\;r_{3}=\frac{k_{12}}{k_{12}+k_{21}}\mathrm{fast}\;\mathrm{increases},\;\mathrm{slow}\;\mathrm{decreases}.$$ (48d)

Case 1 corresponds to Fig. 9(D), while cases 2 and 4 correspond to Fig. 9(C). Case 3 deserves attention because it generates all possible profiles in Fig. 9. The slow relaxation can increase, decrease, or remain constant depending on the relative values of $k_{12}$ and $k_{43},$ and so does the fast relaxation. For example, a small enough value of $k_{21}$ associated with $r_{3}>1$ generates two saturable relaxations that both increase as in Fig. 9(A). On the other hand, a large enough value of $k_{21}$ associated with $r_{3}<1$ generates two saturable relaxations that both decrease as in Fig. 9(B). The linear scheme in Eq. (42) includes both IF and CS, and feature enough flexibility to capture the gamut of functional behaviors observed experimentally as shown in Fig. 10 for 13 different systems.

## XI. DISCUSSION

Assignment of a mechanism of ligand binding from analysis of experimental data has dominated theoretical discussions and experimental investigations for decades. Interpretations of molecular recognition in biology have evolved from the rigid body association of the lock-and-key mechanism to the recognition of conformational transitions through the allosteric concept. This view has further expanded recently to embrace the role of dynamics and molecular ensembles^43,129,130^ unraveled by advanced techniques such as single molecule detection,^132^ NMR,^131,138,180^ and cryo-EM.^133,134^ Several studies have emphasized conformational plasticity and dynamics as key components of macromolecular function, ranging from ligand binding to catalysis.^27,46,181,182^ A distinguishing feature of the interplay between function and dynamics is that macromolecular motion involves communication between segments of the macromolecule that are far apart^183^ and that the timescale for such molecular changes closely matches events such as catalysis^181^ or ligand dissociation.^184^ But no matter how sophisticated our investigation of the properties of a system become, experiments must eventually reconcile information from independent components of our analysis, especially structure and kinetics. This review addressed the role of kinetics in unraveling a mechanism of recognition under conditions most commonly encountered in practice, i.e., the binding of a ligand to a single site of a macromolecular target with the ligand present in excess over the macromolecule and in the absence of changes in ligand linked aggregation. The kinetic properties of these mechanisms are summarized in Table I. Most systems feature a kinetic profile consistent with one saturable relaxation that unequivocally proves the presence of conformational transitions. Whether these transitions precede (CS) or follow (IF), the binding step has been debated for decades.^22,23,53,64,93^ A description of binding in terms of the linkage scheme [Eq. (40)] encompassing IF and CS as special cases^15,24,136,156^ has added value to the debate. The mathematical treatment presented in this review hopefully provided impetus to continue the theoretical investigation of this important area.

**TABLE I.** Basic kinetic schemes and their kinetic properties.

Kinetic Scheme	Relaxations
$E_{\;}\begin{array}{c} k_{on}x\\ ⇄\\ k_{\mathrm{off}} \end{array}EX$	$\alpha (x)=k_{on}x+k_{\mathrm{off}}$
$E\begin{array}{c} k_{on}x\\ ⇄\\ k_{\mathrm{off}} \end{array}EX\begin{array}{c} k_{23}\\ ⇄\\ k_{32} \end{array}E\prime X$	$\begin{array}{c} \alpha_{1}\left(x\right)=\frac{1}{2}\left\{k_{on}x+k_{\mathrm{off}}+k_{23}+k_{32}+\sqrt{{\left(k_{on}x+k_{\mathrm{off}}-k_{23}-k_{32}\right)}^{2}+4{k_{\mathrm{off}}k}_{23}}\right\}\\ \alpha_{2}\left(x\right)=\frac{1}{2}\left\{k_{on}x+k_{\mathrm{off}}+k_{23}+k_{32}-\sqrt{{\left(k_{on}x+k_{\mathrm{off}}-k_{23}-k_{32}\right)}^{2}+4{k_{\mathrm{off}}k}_{23}}\right\}\\ \alpha_{1}(0)=\frac{1}{2}\left\{k_{\mathrm{off}}+k_{23}+k_{32}+\sqrt{{\left(k_{\mathrm{off}}-k_{23}-k_{32}\right)}^{2}+4{k_{\mathrm{off}}k}_{23}}\right\}\\ \alpha_{1}(\infty )\approx k_{on}x\\ \alpha_{2}(0)=\frac{1}{2}\left\{k_{\mathrm{off}}+k_{23}+k_{32}-\sqrt{{\left(k_{\mathrm{off}}-k_{23}-k_{32}\right)}^{2}+4{k_{\mathrm{off}}k}_{23}}\right\}\\ \alpha_{2}(\infty )=k_{23}+k_{32} \end{array}$
${E^{*}}_{\;}\begin{array}{c} k_{12}\\ ⇄\\ k_{21} \end{array}E_{\;}\begin{array}{c} k_{on}x\\ ⇄\\ k_{\mathrm{off}} \end{array}EX$	$\begin{array}{c} \alpha_{1}\left(x\right)=\frac{1}{2}\left\{k_{on}x+k_{\mathrm{off}}+k_{12}+k_{21}+\sqrt{{\left(k_{on}x+k_{\mathrm{off}}-k_{12}-k_{21}\right)}^{2}+4k_{21}k_{on}x}\right\}\\ \alpha_{2}\left(x\right)=\frac{1}{2}\left\{k_{on}x+k_{\mathrm{off}}+k_{12}+k_{21}-\sqrt{{\left(k_{on}x+k_{\mathrm{off}}-k_{12}-k_{21}\right)}^{2}+4k_{21}k_{on}x}\right\}\\ \alpha_{1}\left(0\right)={\mathrm{larger}\;of\;k}_{\mathrm{off}}\;or\;k_{12}+k_{21}\\ \alpha_{1}(\infty )\approx k_{on}x\\ \alpha_{2}(0)={\mathrm{smaller}\;of\;k}_{\mathrm{off}}\;or\;k_{12}+k_{21}\\ \alpha_{2}(\infty )=k_{12} \end{array}$
E*kon*x⇄koff*E*Xk12⇃↾k21 k43⇃↾k34Ekonx⇄koffEX	$\begin{array}{c} \alpha_{1}\left(0\right)=\frac{1}{2}\left\{k_{\mathrm{off}}+k_{\mathrm{off}}^{*}+k_{34}+k_{43}+\sqrt{{\left(k_{\mathrm{off}}+k_{\mathrm{off}}^{*}+k_{34}+k_{43}\right)}^{2}-4\left(k_{\mathrm{off}}k_{\mathrm{off}}^{*}+k_{\mathrm{off}}^{*}k_{34}+k_{\mathrm{off}}k_{43}\right)}\right\}\\ \alpha_{2}(0)=\frac{1}{2}\left\{k_{\mathrm{off}}+k_{\mathrm{off}}^{*}+k_{34}+k_{43}-\sqrt{{\left(k_{\mathrm{off}}+k_{\mathrm{off}}^{*}+k_{34}+k_{43}\right)}^{2}-4\left(k_{\mathrm{off}}k_{\mathrm{off}}^{*}+k_{\mathrm{off}}^{*}k_{34}+k_{\mathrm{off}}k_{43}\right)}\right\}\\ \alpha_{3}(0)=k_{12}+k_{21}\\ \alpha_{1}\left(\infty \right)\approx \mathrm{larger}\;of\;k_{on}x\;or\;k_{on}^{*}x\\ \alpha_{2}\left(\infty \right)\approx \mathrm{smaller}\;of\;k_{on}x\;or\;k_{on}^{*}x\\ \alpha_{3}(\infty )=k_{34}+k_{43} \end{array}$
$E^{*}\begin{array}{c} k_{12}\\ ⇄\\ k_{21} \end{array}E\begin{array}{c} k_{on}x\\ ⇄\\ k_{\mathrm{off}} \end{array}EX\begin{array}{c} k_{34}\\ ⇄\\ k_{43} \end{array}E^{\prime }X$	$\alpha_{2}\left(x\right)=\frac{1}{2}\left\{k_{12}+k_{21}\left(1-f\right)+k_{34}f+k_{43}+\sqrt{{\left[k_{12}+k_{21}\left(1-f\right)-k_{34}f-k_{43}\right]}^{2}+4{k_{21}k}_{34}f(1-f)}\right\}$
Under the rapid equilibrium approximation	$\alpha_{3}\left(x\right)=\frac{1}{2}\left\{k_{12}+k_{21}\left(1-f\right)+k_{34}f+k_{43}-\sqrt{{\left[k_{12}+k_{21}\left(1-f\right)-k_{34}f-k_{43}\right]}^{2}+4{k_{21}k}_{34}f(1-f)}\right\}$
$E^{*}\begin{array}{c} k_{12}\\ ⇄\\ k_{21} \end{array}\langle E|EX\rangle \begin{array}{c} k_{34}\\ ⇄\\ k_{43} \end{array}E^{\prime }X$	$\alpha_{2}\left(0\right)=\mathrm{larger}\;of\;k_{12}+k_{21}\;or\;k_{43}$
	$\alpha_{3}\left(0\right)=\mathrm{smaller}\;of\;k_{12}+k_{21}\;or\;k_{43}$
	$\alpha_{2}\left(\infty \right)=\mathrm{larger}\;of\;k_{12}\;or\;k_{34}+k_{43}$
	$\alpha_{3}\left(\infty \right)=\mathrm{smaller}\;of\;k_{12}\;or\;k_{34}+k_{43}$
	Case 1: $r_{2}=\frac{k_{12}}{k_{12}+k_{21}}\;\mathrm{and}\;r_{3}=\frac{k_{34}+k_{43}}{k_{43}}$ fast ↓, slow ↑ [Fig. 9(D)]
	Case 2: $r_{2}=\frac{k_{12}}{k_{43}}\;\mathrm{and}\;r_{3}=\frac{k_{34}+k_{43}}{k_{12}+k_{21}}$ fast ↑, slow ↓ [Fig. 9(C)]
	Case 3: $r_{2}=\frac{k_{34}+k_{43}}{k_{12}+k_{21}}\;\mathrm{and}\;r_{3}=\frac{k_{12}}{k_{43}}$ fast ↑↓, slow ↑↓ [Figs. 9(A)–(D)]
	Case 4: $r_{2}=\frac{k_{34}+k_{43}}{k_{43}}\;\mathrm{and}\;r_{3}=\frac{k_{12}}{k_{12}+k_{21}}$ fast ↑, slow ↓ [Fig. 9(C)]

The main conclusion of our analysis is that a single saturable relaxation under conditions of excess ligand never disproves *a priori* CS as a mechanism of ligand binding and never makes IF a necessary interpretation of experimental facts.^64,72^ Any experimental dataset compatible with IF can be interpreted with identical mathematical rigor in terms of CS,^59^ which invites a reassessment of conclusions drawn using the rapid equilibrium approximation. The preponderance of systems relaxing to equilibrium with a single saturable relaxation that increases with the ligand concentration has been viewed as dominance of IF as a mechanism of molecular recognition in biology.^4,15,22,28,104^ A similar dominant role for IF has been invoked in enzyme catalysis^33,119^ and as a mechanism to explain the action of irreversible inhibitors,^51,80,83^ without consideration of CS as an alternative interpretation. We have proved that CS is far more versatile then IF as a mechanism of ligand binding and provides a conceptual framework that better fits our current view of macromolecules as dynamic ensembles. If macromolecules are intrinsically plastic in the free form, a correct interpretation of their kinetic properties must reflect such plasticity. CS also generates functional complexity and to a greater extent than IF, even when incorporated into more complex kinetic mechanisms predicting multiple saturable relaxations for the system (Fig. 10). The greater functional contribution of CS to the linkage scheme and the resulting functional asymmetry of a mechanism long recognized as encompassing IF and CS as special cases^15,24,136,156^ deserves attention from future investigations. Biological systems have great complexity, which is increasingly revealed by progress made in experimental techniques. Yet, Ockham's razor reminds us of the value of simple explanations of experimental facts.

## AUTHORS' CONTRIBUTIONS

All authors contributed equally to this manuscript. All authors reviewed the final manuscript.

## Data Availability

The data that support the findings of this study are available from the corresponding author upon reasonable request.
